# Lattice Stabilized and Emission Tunable Pure‐Bromide Quasi‐2D Perovskite for Air‐Processed Blue Light‐Emitting Diodes

**DOI:** 10.1002/advs.202414499

**Published:** 2024-12-06

**Authors:** Yangyang Guo, Penghui Yang, Fan Dong, Huixin Li, Jialiang Gao, Zeyi Cheng, Jiandong Wu, Yadong Xu, Hongyue Wang, Hongqiang Wang

**Affiliations:** ^1^ State Key Laboratory of Solidification Processing Center for Nano Energy Materials School of Materials Science and Engineering Northwestern Polytechnical University and Shaanxi Joint Laboratory of Graphene (NPU) Xi'an 710072 P. R. China

**Keywords:** air‐processed light‐emitting diodes, deep blue emission, interstitial doping, pure‐Br quasi‐2D perovskite

## Abstract

Realizing air‐processed blue halide perovskite films with tailored emission is significant for promoting the commercialization of perovskite light‐emitting diodes (PeLEDs). However, the intrinsically inferior thermodynamic stability and laborious crystallization kinetics control under humidity interference limit the fabrication of blue perovskite emitters in ambient air. Here, air‐processed pure‐bromide quasi‐2D blue perovskite films are achieved with stabilized lattice and tunable emission by interstitial doping of trivalent metallic cations. This strategy improves the formation energy of the perovskite lattice, promotes energy transfer between different n phases, and suppresses intrinsic electron‐phonon coupling in the perovskite films. The emission‐controllable blue PeLEDs are fabricated in ambient air for the first time. The champion deep blue PeLED shows maximum external quantum efficiency (EQE) of 2.05% and luminance of 246.56 cd m^−2^, which are comparable to the state‐of‐the‐art of similar devices fabricated in glovebox. The work pioneers a simple method of electronic structure engineering to tune the emission of air‐processed blue perovskite, breaking the limitations of thermodynamic stability and crystallization kinetics control of perovskite in ambient air.

## Graphical abstract

The study presents trivalent interstitial ions‐doped blue perovskite films and PeLEDs under ambient condition (≈25% RH and 20 °C), showing tunable PL emission (from sky blue to deep blue) with impressive stability, owing to modulated crystallization kinetics and enhanced the formation of the n = 3 phase, as well as suppressed electron–phonon coupling in the perovskite films.

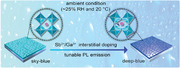

## 1 Introduction

Metal halide perovskites have emerged as highly promising emissive materials for light‐emitting diodes (LEDs) in application of display.^[^ ^1^ ^]^ The electronic structure of perovskite could be easily tailored by component engineering (mixing halides Br/Cl)^[^ ^2^ ^]^ or dimensional engineering (owing the quantum confinement in perovskite quantum wells or quantum dots),^[^ ^3^ ^]^ those strategies have been applied to effectively tune the emission peak wavelength of blue perovskite emitters. In particular, the incorporation of component engineering and dimensional engineering has become the primary method to fabricate efficient blue perovskite LEDs (PeLEDs) by mixing chloride ions in pure‐bromide (pure‐Br) quasi‐2D blue perovskite.^[^ ^4^ ^]^ The great advances of the record external quantum efficiency (EQE) of blue PeLEDs beyond over 20% has been achieved.^[^ ^5^ ^]^ However, the denounced stability issue still remains in the inherently ionic perovskite lattice, the mixing halide perovskite usually exhibits severe halogen segregation.^[^ ^6^ ^]^

Since the air‐processed perovskite techniques have been considered as an inevitable necessity for the further proceeding perovskite optoelectronics in commercialization,^[^ ^7^ ^]^ the mixing halide blue perovskite apparently do not comply to the air‐processed procedures. The severe halogen segregation will be even more pronounced under ambient conditions due to the exacerbated chemical and structural disorder,^[^ ^8^ ^]^ lower halide ion migration barriers, and anisotropic crystallization^[^ ^9^ ^]^ with the presence of chloride ions in the blue perovskite. Furthermore, the low solubility^[^ ^10^ ^]^ and high hygroscopicity^[^ ^11^ ^]^ of chloride precursors remarkably limit both the spectral tunability and long‐term stability of the air‐processed blue perovskite emitters. In contrast, pure‐Br perovskite has a much more stable lattice than that of mixing halide perovskite;^[^ ^12^ ^]^ computational studies have demonstrated that CsPbBr_3_ exhibits a more stable lattice structure than CsPbCl_3_ or their mixing halide system due to the more compatible ionic radii.^[^ ^13^ ^]^ Although perovskite nanocrystals exhibit enough stability to against the stimuli of ambient conditions due to the organic ligands on the surface,^[^ ^14^ ^]^ the air‐processed blue PeLEDs based on perovskite nanocrystals have not yet been reported. So far, pure‐Br quasi‐2D blue perovskite is an expected candidate for realizing the fabrication of air‐processed blue perovskite emitter and PeLEDs. However, only a few works successfully achieve the air‐processed blue perovskite and PeLEDs by tuning pure‐Br quasi‐2D perovskite crystallization kinetics based on the absorbed moisture control and the substrate surface modification.^[^ ^15^ ^]^ These preliminary achievements are still far from the requirements of tunable emission and manipulatable stability.

Although the record EQE of blue PeLEDs based on pure‐Br quasi‐2D blue perovskite fabricated in glovebox could reach to 16.07%,^[^ ^16^ ^]^ the vulnerable perovskite lattice allows the dynamic reconstruction and regrowth between different n phases (the perovskite quantum wells with different layers of octahedral) of pure‐Br quasi‐2D blue perovskite during spin‐coating and annealing under the stress of humidity in ambient air, resulting in uncontrollable PL emission and low photoluminescence quantum yield (PLQY).^[^ ^7^, ^17^ ^]^ Fundamentally improving the thermodynamic stability of perovskite lattice to increase the formation energy of certain n phases^[^ ^18^ ^]^ would thus be consequently promising for eradicating the humidity induced PL emission variation and PLQY deterioration of air‐processed blue perovskite emitters with high manipulability in the wide emission window. However, in the state‐of‐the‐art, rising the intrinsic formation energy of the perovskite lattice with tailorable electronic structure for emission control is a formidable challenge.

Herein, we demonstrate an efficient strategy for electronic structural engineering of metal halide perovskite based on trivalent metallic cation interstitial doping, which effectively tunes the PL emission of air‐processed pure‐Br quasi‐2D blue perovskite film in the range from sky blue to deep blue (482 nm to 468 nm). Exemplified by the interstitial doping of Sb^3+^ and Ga^3+^, the crystallization kinetics of air‐processed perovskite are well modulated, and the presence of interstitial ions increases the formation energy of the n = 3 phase and prevents the migration of bromine ions in the perovskite lattice, which remarkably improves the stability of perovskite under the stress of heating and humidity. Furthermore, the Ga^3+^‐doped perovskite films maintained blue PL emission with slight red shifting of a few nanometers after 72 h of heating at 70 °C or exposure to air with 20–25% RH for 120 h. It was also found that the interstitial ions improve the energy transfer between different n phases and suppress the electron‐phonon coupling in the blue perovskite films. The resultant air‐processed blue PeLEDs show widely tunable emission in the range of sky blue to deep blue, with maximum external quantum efficiencies (EQEs) of 3.02%, 2.39%, and 2.05% at emission peaks of 480 nm (sky blue), 474 nm (true blue), and 468 nm (deep blue), respectively.

## 2 Results and Discussion

### 2.1 Air‐Processed Blue Quasi‐2D Perovskite Films with Tunable PL Emission

**Figure 1:**
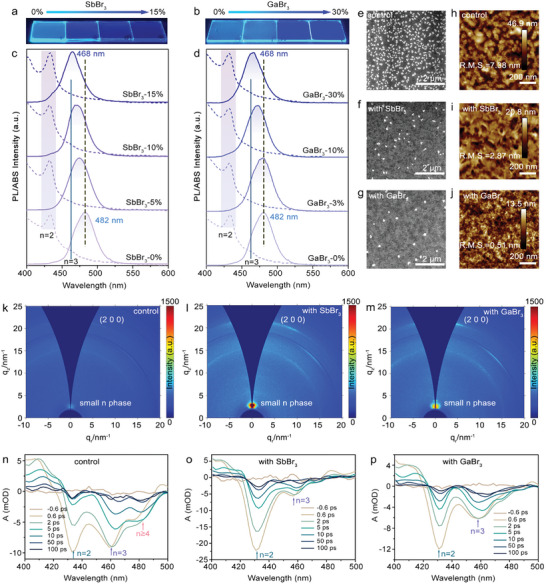
Properties of perovskite films incorporated with trivalent metallic cations. Photograph of perovskite films incorporated with a) SbBr_3_ and b) GaBr_3_ under UV light. PL and corresponding absorption spectra of quasi‐2D perovskite films with c) SbBr_3_ and d) GaBr_3_. e–g) SEM images and h–j) corresponding AFM images of perovskite films. k–m) 2D GIWAXS profiles and n–p) TAS spectra of the control, SbBr_3_‐treated, and GaBr_3_‐treated perovskite films.

The desired quasi‐2D PEA_2_(CsPbBr_3_)_2_PbBr_4_ perovskite films with different ratios of XBr_3_ (X = Sb or Ga) were obtained through the spin‐coating process in ambient conditions (≈ 25% RH and 20 °C). Details about the film fabrication can be found in Supporting Information. The images under UV light, absorption, and PL spectra of the films are shown in **Figure** **1**a–d. The control perovskite film exhibits PL emission in sky blue with a peak wavelength of ≈482 nm and an excitonic absorption peak at 430 nm. The addition of SbBr_3_ and GaBr_3_ both lead to a blueshift in the PL emission of the perovskite films. The PL peak gradually shifts and reaches a deep blue of 468 nm when the ratios of SbBr_3_ and GaBr_3_ are 15% and 30% relative to PbBr_2_, respectively. As seen in Figure 1c,d, all samples exhibit a prominent excitonic absorption at 430 nm, attributed to n = 2 low‐dimensional domains. In the control sample, an additional peak at 460 nm corresponds to n = 3 domains. Moreover, a distinct band‐edge absorption signal in the 475–500 nm range, with a notable increase in intensity, is attributed to the n ≥ 4 phase. For the SbBr_3_‐ and GaBr_3_‐treated films, the absorption edge shifts to the 450–475 nm range, corresponding to n = 3 absorption. These variations indicate that the phase distribution of the quasi‐2D perovskite films is altered by the introduction of trivalent ions. Figure 1e–j shows the surface morphology of the films, and it can be observed that many precursor compounds are segregated on the surface of the control film, leading to a high surface roughness of 7.38 nm. The presence of SbBr_3_ and GaBr_3_ can remarkably suppress such segregation and reduce the surface roughness to 2.87 nm and 0.51 nm for the films with SbBr_3_ and GaBr_3_, respectively. The precipitation of CsBr as white precursor compounds has been confirmed by transmission electron microscopy (TEM) and energy dispersive spectroscopy (EDS), as shown in Figure S1 (Supporting Information). Previous studies have indicated that CsBr, a Cs‐based precursor, exhibits poor solubility in common aprotic solvents such as DMSO or DMF,^[^ ^19^ ^]^ which leads to a poorly controllable crystallization process. During air‐processed fabrication, ambient moisture accelerates the crystallization of CsBr, resulting in the formation of numerous white particles within the perovskite film (Figure 1e) and consequently high surface roughness. However, the incorporation of SbBr_3_ or GaBr_3_ significantly reduces the precipitation of CsBr, resulting in a smoother film morphology. The PLQY of these perovskite films shown in Figure S2 (Supporting Information) indicates that even though these films with SbBr_3_ and GaBr_3_ exhibit PL emission at a much shorter wavelength compared with the control film, they still have a higher PLQY overall.

We conducted grazing‐incidence wide‐angle X‐ray scattering (GIWAXS) measurements to investigate the crystal structure of these perovskite films, as shown in Figure 1k–m. All films show typical perovskite diffraction characteristics at ≈21 nm^−1^, corresponding to the (200) crystal planes. The GIWAXS patterns of the control films manifested as isotropic diffraction rings, suggesting that perovskite crystallites are randomly oriented.^[^ ^20^ ^]^ By contrast, the scattering intensity of both Sb^3+^‐treated and Ga^3+^‐treated perovskite films increased, and the concentration of diffraction intensity was centered at some hot spots, indicating a preferential orientation of trivalent cation‐treated perovskites along the surface normal direction (Figure 1e–j).^[^ ^21^ ^]^ Additionally, the intensity of the diffraction peak present at q_z_ ≈ 3.5 nm^−1^ was significantly increased in Sb^3+^‐treated and Ga^3+^‐treated perovskite films compared to the control film, signifying the generation of low‐n phases in the quasi‐2D perovskite system.

Since the PL emission of quasi‐2D perovskite films is strongly determined by the n‐phase distribution, we conducted femtosecond transient absorption (TA) measurements on the films, as shown in Figure 1n–p. The control film shows ground‐state bleaching signals of n = 2, n = 3, and n = 4. The intensity of the n = 4 phase decreases or even disappears with the addition of SbBr_3_ and GaBr_3_. Therefore, the tunable PL emission of the perovskite films could be attributed to n‐phase distribution control upon the addition of SbBr_3_ and GaBr_3_. The n‐phase distribution and kinetic decay of these films are shown in Figure S3 (Supporting Information). The ratio of n phases of n = 3 is reduced in the films added with SbBr_3_ and GaBr_3_ compared to that of the control film, and the characteristic energy funnel processes from small n‐phase to large n‐phase can be clearly observed in the kinetic decay curves. The addition of SbBr_3_ or GaBr_3_ introduces extra Br^−^ into the precursor, which could change the stoichiometry of perovskite and thus the n‐phase distribution. To investigate if extra Br^−^ matters, we fabricated perovskite films by adding extra PbBr_2_, which however leads to red shift of PL emission (Figure S4, Supporting Information) and has no significant influence on the surface morphology of the perovskite films (Figure S5, Supporting Information). Meanwhile, to exclude the effect of excess Pb ions, we fabricated perovskite films by incorporating LaBr_3_. As shown in Figure S6 (Supporting Information), the emission peak of the control film exhibited no significant difference compared to the LaBr₃‐treated films. It can thus be concluded that trivalent ions of Sb^3+^ and Ga^3+^ dominate the modulation of PL emission, n‐phase distribution, and morphology of the perovskite films.

We have also fabricated SbBr_3_‐treated and GaBr_3_‐treated perovskite films in glovebox under an inert gas atmosphere, with the corresponding PL spectra shown in Figure S7 (Supporting Information). Increasing the concentrations of SbBr_3_ and GaBr_3_ in glovebox shifts the PL emission of perovskite films gradually from ≈480 nm (sky blue) to 468 nm (deep blue). This shift is consistent with the blue‐shifted PL spectra observed in air‐processed perovskite films with equivalent concentrations of trivalent ions. The data indicate that ambient conditions neither hindered nor facilitated the fabrication of perovskite films with tunable spectra, confirming that Sb^3+^ and Ga^3+^ ions are responsible for the tunable PL emission of perovskite films, regardless of ambient or inert environments.

### 2.2 Interstitial Doping of the Trivalent Metal Ions in the Perovskite

**Figure 2:**
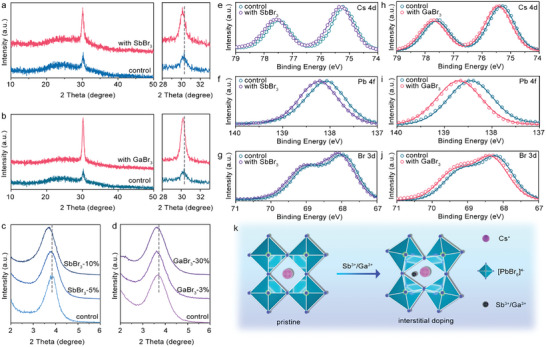
Interstitial doping of the trivalent metal ions in the perovskite. XRD patterns of perovskite films doped with a) SbBr_3_ and b) GaBr_3_. Small‐angle XRD patterns of perovskite films doped with c) SbBr_3_ and d) GaBr_3_, with the peak at 2θ = 3.9° corresponding to n = 2 of quasi‐2D perovskite. XPS signals for Cs 4d, Pb 4f, and Br 3d of e–g) SbBr_3_‐doped perovskite films and h–j) GaBr_3_‐doped perovskite films. k) Illustration of CsPbBr_3_ perovskite lattices without and with Sb^3+^ or Ga^3+^ at the interstitial site.

XRD data of the perovskite films are shown in **Figure** **2**a,b, where the Sb^3+^ and Ga^3+^ ions‐doped films show a typical quasi‐2D perovskite structure with a signal of (200) planes at 30.31°. The diffraction intensity of Sb^3+^ and Ga^3+^ ion‐doped films showed a significant increase compared with that of the control film, confirming the improved crystallization after the doping of trivalent metal cations. Meanwhile, the diffraction peak shifted to a smaller angle in the Sb^3+^ and Ga^3+^ ion‐doped films. Small‐angle XRD data of these films are shown in Figure 2c,d, where a diffraction peak located at 3.9° can be assigned to the n = 2 phase of the quasi‐2D perovskite.^[^ ^10^ ^]^ This diffraction peak also gradually shifted to a smaller angle with the increased doping concentration of these trivalent metal cations. As depicted in Figure S8 (Supporting Information), the trivalent metal cations of Sb^3+^ and Ga^3+^ are not allowed to dope in the perovskite lattice by substituting the A site cation of Cs^+^, due to the fact that the trivalent metal cations have much smaller effective ionic radii that cannot sustain the perovskite lattice with a reasonable tolerance factor. Meanwhile, if the doping of the trivalent metal cations substitutes the B sites of Pb in the perovskite lattice, the lattice constant would become smaller because the effective ionic radii of Sb^3+^ and Ga^3+^ are smaller than Pb^2+^ (Figure S8, Supporting Information). Thus, the corresponding XRD peaks would shift to a larger degree of 2θ. However, in our work, a monotonic shift of the XRD peaks to smaller angles with an increased concentration of the trivalent ion was observed, indicating that the Sb^3+^ and Ga^3+^‐doped films experienced tensile strain induced by the incorporation of the trivalent metal cations.

To investigate how trivalent ions affect the lattice structure of quasi‐2D perovskite films, we carried out X‐ray photoelectron spectroscopy (XPS) measurements to identify the chemical states of Sb^3+^ and Ga^3+^ in the perovskite films. As shown in Figure S9 (Supporting Information), the binding energy peaks located at 540 and 397 eV correspond to the Sb 4d_2/3_ core level and Ga LMN signal, respectively, which confirms the existence of trivalent metal cations in the perovskite films. Figure 2e–j shows the XPS spectra for all relevant elements, including Cs, Pb, and Br, of both control and doped films. The difference in the peaks for the control film between Figure 2f,i arises from two independent XPS measurements. Notably, in the Sb^3+^ and Ga^3+^‐doped perovskite films, the XPS peaks for all elements were shifted to higher binding energy compared to those of the control film, indicating a strong interaction of the Sb^3+^ or Ga^3+^ with the perovskite lattice. Therefore, it is considered that the addition of Sb^3+^ and Ga^3+^ generates interstitial doping instead of substituting A or B sites, as shown in Figure 2k. The interstitial doping of Sb^3+^ and Ga^3+^ ions induce tensile strain on the perovskite lattice, enlarging the lattice constant and shifting the XRD signal to a smaller angle.^[^ ^4b^ ^]^ Additionally, the trivalent interstitial cations generate a local positive electrical field, causing strong interactions with Cs, Pb, and Br ions in the perovskite lattice,^[^ ^22^ ^]^ which leads to a shift in their XPS signals.

### 2.3 Crystallization Kinetic of Interstitial Ions Doped‐Perovskite Films

**Figure 3:**
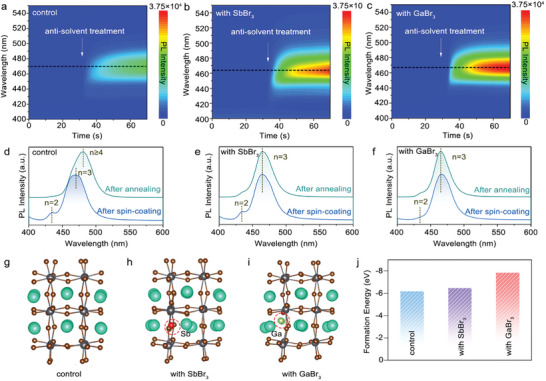
Modulation of crystallization kinetics via interstitial ion doping. Pseudo‐color plot of in‐situ PL spectra of perovskite films during the spin‐coating process under ambient air: a) control, b) Sb^3+^‐treated perovskite films, and c) Ga^3+^‐treated perovskite films. The PL spectra of d) control, e) Sb^3+^‐treated perovskite films, and f) Ga^3+^‐treated perovskite films before and after annealing stages. DFT results of formation energy for low dimensional species (n = 3) based on (g) control, h) Sb^3+^‐treated, and i) Ga^3+^‐treated perovskite systems. j) DFT results comparing the three types of perovskite films (control, Sb^3+^‐treated, and Ga^3+^‐treated).

The perovskite lattice is very sensitive to moisture, which significantly interferes with the crystallization of the perovskite films under ambient conditions.^[^ ^23^ ^]^ We conducted in‐situ PL measurements to monitor the crystallization kinetics of the perovskite films during spin‐coating under air (≈ 25% RH and 20 °C). As shown in **Figure** **3**a–c, the PL emission of all perovskite films is triggered at the same moment of anti‐solvent treatment; the control film shows PL emission with a peak wavelength of ≈470 nm, and the trivalent interstitial ion‐doped perovskite films show PL emission with a peak in the range of ≈460–470 nm. All in‐situ PL measurement conditions were strictly controlled to be the same for all films to guarantee the PL intensity comparison. The trivalent interstitial ion‐doped perovskite films exhibit much higher PL intensity compared with the control film after anti‐solvent treatment. Furthermore, by increasing the concentration of the trivalent interstitial ions, the PL intensity increases in these perovskite films during spin‐coating (Figure S10, Supporting Information). Then, these perovskite films were annealed at 80 °C for 1 min. The PL spectra comparison of these films after spin‐coating and after annealing are shown in Figure 3d–f. The control film shows a remarkable PL red‐shift after annealing, while the trivalent interstitial ion‐doped perovskite films show stationary PL spectra after annealing, indicating that the trivalent ions bring improved stability to the n phase distribution, particularly the small n phases, which are robust during annealing to sustain the blue emission.

Because the n = 3 phase dominates the blue emission of our perovskite film in the wavelength range of 460–480 nm, and to study the origin of the improved stability of the interstitial ion‐doped perovskite films, we then conducted density functional theory (DFT) calculations to study the formation energy of the n = 3 phase in the perovskite films (Figure 3g–j). The Sb^3+^ and Ga^3+^‐containing perovskites present formation energy of ‐6.482 eV and ‐7.861 eV for the low‐dimensional species of the n = 3 phase, respectively, which are both higher than that of the control perovskites (−6.194 eV). The higher formation energy implies higher stability of the n = 3 phase against heating and moisture. The result of higher formation energy in the n = 3 phase is also consistent with the reduced ratio of n = 3 phase in the perovskite films in TA measurement (Figure 1n–p). Therefore, the interstitial ion doping totally suppresses the formation of the n = 4 phase and partially suppresses the formation of the n = 3 phase because of the enhanced formation energy. This not only leads to blue‐shifted PL emission from sky blue to deep blue but also gives rise to the improved stability of the perovskite films fabricated in air.

Furthermore, it has been reported that the local positive electric field from the trivalent interstitial doped ions could remarkably enhance the stability of the perovskite lattice by binding halogen ions to reduce their migration and corresponding defects.^[^ ^24^ ^]^ Time‐resolved photoluminescence (TRPL) and space‐charge‐limited current (SCLC) measurements were carried out (Figures S11 and S12, and Tables S1 and S2, Supporting Information). Both results show that the perovskite films incorporated with Sb^3+^ and Ga^3+^ exhibited reduced trap defects. We attributed the decreased defect density to the fact that Sb^3+^ and Ga^3+^ ions, with their three positive charges, were more likely to restrict the motion of negative bromine ions due to the local electrostatic attractive force. The impeded bromine ion migration facilitates the elimination of halogen vacancies, thereby reducing the deep trap states in the center of the bandgap.^[^ ^24^ ^]^ Meanwhile, trivalent interstitial cations are likely to interact with negatively charged defects in the perovskite film to achieve local charge balance, further minimizing the trap states in the perovskite films.

### 2.4 Stability Test and Carrier Dynamic Study of the Interstitial‐Doped Perovskite Films

**Figure 4:**
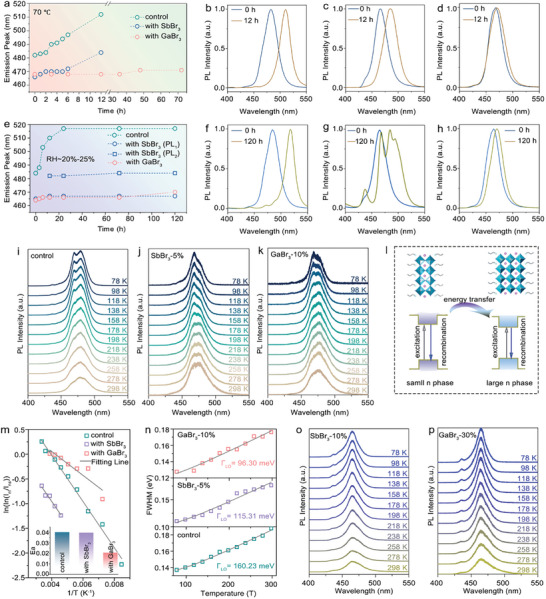
Stability enhancements and reduced energy barrier in interstitial‐doped perovskite films. Thermal stability test of interstitial doped‐perovskite films heated at 70 °C for 72 h: a) the evolution of PL emission peaks as function of time and b–d) the corresponding PL spectra. Humidity stability test of interstitial doped‐perovskite films in an ambient environment with relative humidity of 20–25% for 120 h: e) the evolution of PL emission peak as function of time and f–h) the corresponding PL spectra. The temperature‐dependent PL spectra of i) control, J) 5% SbBr_3_‐doped, and k) 10% GaBr_3_‐doped perovskite films measured at temperatures ranging from 78 to 298 K. l) Schematics depicting the energy transfer process from small n phase to large n phase. m) Arrhenius equation fitting through integrated PL intensity of large n phase over small n phase $\ln(\ln(I_{n_{l}}/I_{n_{s}}))$ as a function of temperature. Inset: histogram of active energy (Ea) for the control and interstitial doped‐perovskite films. n) Boson model fitting through FWHM as a function of temperature for the control and interstitial doped‐perovskite films. The temperature‐dependent PL spectra of (o) 10% SbBr_3_‐doped and p) 30% GaBr_3_‐doped perovskite films measured at temperatures ranging from 78 to 298 K.

We further tested the thermal stability of the films by heating them on a hot plate at 70 °C for 72 h. The PL spectra evolution is shown in **Figure** **4**a–d and Figures S13 and S14 (Supporting Information). The control film and the Sb^3+^‐doped film show significant redshifts in PL emission, from ≈480 to ≈500 nm and from ≈466 to ≈485 nm, respectively, while the GaBr_3_‐doped film exhibits robust stability against heat, showing only a negligible red shift in PL emission. The improved stability was also confirmed by the corresponding absorption spectra of these films (Figure S15, Supporting Information).

The humidity stability of the films was tested in an ambient environment with a relative humidity of 20–25% at room temperature. The results of the spectra are shown in Figure 4e–h and Figures S16–S19 (Supporting Information). The control film shows a remarkable red shift in PL emission from ≈480 to ≈510 nm after being exposed to air for 120 h. Although the Sb^3+^‐doped film remains blue‐emitting after exposure to air for 120 h (Figure S16, Supporting Information), the PL spectra exhibit multi‐peaks located at 437, 467, and 484 nm, respectively (Figure 4e; Figure S17, Supporting Information). Those multi‐peaks correspond to the broadened n phase distribution of n = 2, n = 3, and n ≥ 4, respectively. Moreover, the multiple peaks in PL spectra are stationary, even with an extended exposure time beyond 120 h (Figure 4e). Compared with the control and Sb^3+^‐doped films, the Ga^3+^‐doped film shows impressive stability under the same ambient condition, with only a slight red shift of a few nanometers in PL emission (Figure 4e), and its full width at half maximum (FWHM) remained almost constant (Figure S18, Supporting Information). A similar trend was also observed in the absorption spectra of these films, as shown in Figure S19 (Supporting Information). For the control and Sb^3+^‐doped perovskite films, the excitonic absorption peak assigned to n = 3 species was observed, and its intensity gradually increased with the prolongation of the test time, reflecting the formation of the n = 3 phase. However, throughout the entire test process, a significant increase in absorption peaks was not observed in the Ga^3+^‐doped film, further demonstrating that the n phase distribution in the Ga^3+^‐incorporated perovskite film remained unchanged.

Apparently, Sb^3+^ and Ga^3+^ ions can sustain the n phase distribution of the perovskite films during spin‐coating and annealing, allowing for blue emission when compared with the control film (Figure 3a–f). However, Ga^3+^‐doped films exhibit much higher stability under the stress of heating or humidity. This impressive stability of Ga^3+^ ions‐doped blue perovskite films is probably attributed to the smaller radius of Ga^3+^ ions compared to that of Sb^3+^ ions, which facilitates effective interstitial doping of Ga^3+^ into the perovskite lattice, as well as the reduced defect density due to the higher electrostatic interaction between Ga^3+^ ions and bromine ions.^[^ ^24^ ^]^

Since carrier dynamics are of significance for understanding the optical properties and even stability of perovskite films, we carried out temperature‐dependent PL measurements to study how these interstitial ions affect carrier kinetic in the perovskite films. To avoid variations due to different band structures of perovskite, we explored the PL evolution with temperature in these perovskite films, which exhibit PL emission at a similar wavelength (≈475 nm), as shown in Figure 4i–k. A small and ignorable peak appears at a wavelength of ≈430 nm, which is assigned to the n = 2 phase at low temperature for all films. By decreasing the temperature from 298 to 78 K, the PL spectrum of the control film splits into two peaks, while the Sb^3+^‐doped film and Ga^3+^‐doped film exhibit blue‐shifted PL emission rather than splitting. More specifically, the shoulder peak located at a short wavelength gradually increases in intensity, becoming the main peak as the temperature decreases. Consequently, the energy transfer process from small n phase to large n phase is smoother in the Sb^3+^‐ and Ga^3+^‐ion‐doped films (Figure 4l), even though the activity of the energy funnel effect could be reduced in the quasi‐2D perovskite films at low temperatures. To quantitatively analyze the energy transfer process in these films, we fitted the temperature‐dependent PL spectra and extracted the active energy (E_a_) with an Arrhenius equation to describe the energy barrier between two n phase in the perovskite films (Figure S20, Supporting Information; Figure 4m). The values of E_a_ in Sb^3+^‐ and Ga^3+^‐doped films are smaller than those of the control film, indicating a smaller energy barrier between the different n phases in the doped films.

Besides, it has been reported that the small n phase of perovskite generally exhibits strong electron‐phonon coupling, which induces broadened linewidth, PL shifting, and non‐radiative recombination.^[^ ^25^ ^]^ We also studied the electron‐phonon coupling in our perovskite films by fitting the temperature‐dependent PL linewidth using the Boson model. As shown in Figure 4n and Table S3 (Supporting Information), the extracted Γ_LO_ (the exciton longitudinal optical phonon coupling coefficient) is estimated to be 160.23, 115.31, and 96.30 meV for the control film, Sb^3+^‐treated film, and Ga^3+^‐treated film, respectively. Therefore, trivalent interstitial ions could suppress the electron‐phonon coupling in the perovskite films, probably because the trivalent interstitial ions‐induced tensile strain increases the stiffness of the soft perovskite lattice.^[^ ^26^ ^]^ Suppression of electron‐phonon coupling facilitates efficient carrier transport, which is consistent with the observed reduction in the active energy of E_a_. Further increasing the ratio of the trivalent interstitial ions of Sb^3+^ and Ga^3+^, which shifts the PL emission of the perovskite films to deep blue and reduces the linewidth of the PL spectra, as shown in Figure 4o,p. We found that the multi‐peak emission in the range of 460–470 nm is completely suppressed with decreased temperature. This further confirms that the trivalent interstitial ions could achieve a narrow n phase distribution and suppression of electron‐phonon coupling in the perovskite films.

### 2.5 Air‐Processed PeLEDs with Deep Blue Emission

**Figure 5:**
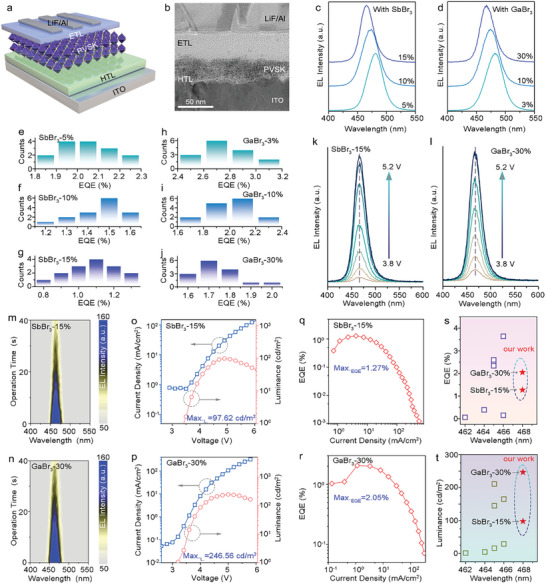
The performance of air‐processed blue PeLEDs with various trivalent metal ions contents. a) Schematic diagram and b) cross‐sectional TEM image of air‐processed blue PeLEDs. EL spectra of the fabricated PeLEDs with varying concentrations of c) SbBr_3_ and d) GaBr_3_. Histograms showing the peak EQE of devices treated with e) 5% SbBr_3_, f) 10% SbBr_3_, g) 15% SbBr_3_, h) 3% GaBr_3_, i) 10% GaBr_3_, and j) 30% GaBr_3_. EL spectra of deep blue PeLEDs based on k) 15% SbBr_3_ and l) 30% GaBr_3_ at different bias voltages. EL contour mapping under different operation time for deep blue PeLEDs with m) 15% SbBr_3_ and n) 30% GaBr_3_. Current density‐voltage and luminance‐voltage characteristics of deep blue PeLEDs with o) 15% SbBr_3_ and p) 30% GaBr_3_. EQE‐current density curves for deep blue PeLEDs with q) 15% SbBr_3_ and r) 30% GaBr_3_. (s and t) Comparison of our work with previous deep blue PeLED results (fabricated in N_2_ glovebox) with EL emissions ranging from 460 to 470 nm.

The PeLEDs were fabricated under ambient conditions with a structure of glass/indium tin oxide (ITO)/Polyvinylpyrrolidone (PVP)/Perovskite/tris(1‐phenyl‐1H‐benzimidazol‐2‐yl) benzene (TBPi)/ LiF/Al, as illustrated in **Figure** **5**a. The thermal evaporations of TPBi and the Al electrodes were conducted in vacuum deposition equipment, while the other fabrication processes were carried out under ambient conditions (≈25 °C, ≈25% RH). Each functional layer could be clearly observed in the cross‐sectional transmission electron microscopy (TEM) image shown in Figure 5b. The control pure‐Br quasi‐2D PeLEDs exhibited sky blue emission (482 nm) with a maximum luminance of 534.66 cd m^−2^ and a maximum EQE of 1.92% (Figure S21, Supporting Information). By increasing the doping concentration of Sb^3+^ or Ga^3+^ ions, the EL emission of the PeLEDs could be tailored from sky blue (480 nm for 5% of Sb^3+^ or 3% of Ga^3+^ ions), true blue (474 nm for 10% of Sb^3+^ or 10% of Ga^3+^ ions), to deep blue (468 nm for 15% of Sb^3+^ or 30% of Ga^3+^ ions), as shown in Figure 5c,d. All air‐processed pure‐Br quasi‐2D blue PeLEDs show good reproducibility with average EQE of 2.08% for sky blue of SbBr_3_‐based PeLEDs, 1.42% for true blue of SbBr_3_‐based PeLEDs, 1.08% for deep blue of SbBr_3_‐based PeLEDs, as well as 2.76% for sky blue of GaBr_3_‐based PeLEDs, 2.04% for true blue of GaBr_3_‐based PeLEDs, 1.73% for deep blue of GaBr_3_‐based PeLEDs (Figure 5e–j; Figures S22–S25, Supporting Information).

The champion devices of SbBr_3_ and GaBr_3_‐based pure‐Br quasi‐2D PeLEDs show deep blue emission at 468 nm with corresponding CIE coordinates of (0.13, 0.06), which is facilitated to the application in display (Figure S26, Supporting Information). These pure‐Br quasi‐2D deep blue PeLEDs exhibit stable EL spectra under increased voltage, owing to trivalent interstitial doping ions that suppress the migration of bromine to enhance the stability of perovskite lattice under electric field (Figure 5k,l). We then conducted the *T*_50_ lifetime measurements (the time when the luminance drops to 50% of its initial value) of the devices under similar initial luminance and operating conditions (Figure S27, Supporting Information). The SbBr_3_ and GaBr_3_‐based pure‐Br quasi‐2D deep blue PeLEDs show operational lifetime of 28 and 42 s, respectively. Both devices exhibit excellent EL spectral stability (Figure 5m,n) compared to similar PeLEDs fabricated in an N_2_ glovebox. Figure 5o–r depicts the current density‐voltage‐luminance (J‐V‐L) and current density‐EQE (J‐EQE) curves. The pure‐Br quasi‐2D deep blue PeLEDs incorporated with SbBr_3_ exhibited a maximum luminance of 97.62 cd m^−2^ and a maximum EQE of 1.27% (Figure 5o,p), while the peak EQE of the GaBr_3_‐treated pure‐Br quasi‐2D deep blue device reaches to 2.05%, with a maximum luminance of 246.56 cd m^−2^ (Figure 5q,r). Since the fundamental issue of halide segregation in mixed chloride‐bromide perovskites, and air‐processed deep blue PeLEDs have only achieved on the pure‐Br quasi‐2D perovskite system, we made the comparison of reported deep blue PeLEDs based on pure‐Br quasi‐2D perovskite fabricated in glove box and ambient air in this work. As summarized in Figure 5s, the EQE of the air‐processed pure‐Br quasi‐2D deep blue PeLEDs in this work is ranked at the top in the state‐of‐the‐art for pure‐Br quasi‐2D deep blue PeLEDs.^[^ ^27^ ^]^ Notably, the GaBr_3_‐treated champion pure‐Br quasi‐2D deep blue PeLED exhibited a maximum luminance of 246.56 cd m^−2^, which is the top value among similar deep blue PeLEDs, as shown in Figure 5t.^[^ ^27^ ^]^

## 3 Conclusion

In summary, we demonstrated a strategy involving the trivalent interstitial ions of Sb^3+^ and Ga^3+^ doping, enabling the air‐processed fabrication of pure‐Br quasi‐2D perovskite films with controllable emission ranging widely from sky blue to deep blue (482–468nm). The resulting blue perovskite films exhibit improved crystallinity and enhanced stability against humidity and heating in ambient air. This improvement is due to the trivalent interstitial ions‐induced modulation of crystallization kinetic and the enhanced formation energy of the n = 3 phase for blue emission. Moreover, the presence of trivalent interstitial ions benefits the energy transfer from small n phases to larger ones and suppresses the electron‐phonon coupling in the blue perovskite films, which enhances the light efficiency of the films and devices. For the first time, emission‐controllable blue PeLEDs (from sky blue to deep blue) were fabricated in ambient air, showing good stability and reproducibility. The air‐processed deep blue PeLEDs yield a maximum EQE of 2.05% and a maximum luminance of 246.56 cd m^−2^, which are comparable to the state‐of‐the‐art for pure‐Br quasi‐2D PeLEDs with deep blue emission fabricated in N_2_ glovebox. Our work provides a strategy that breaks the limitation of blue PeLEDs fabrication requiring protection from inert atmosphere in the glovebox, which opens avenues for the construction of air‐processed PeLEDs and paves the way to tackle the challenges of commercializing PeLEDs for display application.

## Conflict of Interest

The authors declare no conflict of interest.

## Supporting information



Supporting Information

## Data Availability

The data that support the findings of this study are available from the corresponding author upon reasonable request.
