# Supplementary material for: Lattice Stabilized and Emission Tunable Pure‐Bromide Quasi‐2D Perovskite for Air‐Processed Blue Light‐Emitting Diodes
Source: Adv Sci (Weinh). 2024 Dec 6;12(5):2414499. doi: 10.1002/advs.202414499 (PMC11791954; doi:10.1002/advs.202414499)
Supplement: Supplementary file 1 — Supporting Information [file ADVS-12-2414499-s001.docx]

**Supporting Information**

**Lattice Stabilized and Emission Tunable Pure-Bromide Quasi-2D Perovskite for Air-Processed Blue Light-Emitting Diodes**

Yangyang Guo,^[a]^ Penghui Yang,^[a]^ Fan Dong,^[a]^ Huixin Li,^[a]^ Jialiang Gao,^[a]^ Zeyi Cheng,^[a]^ Jiandong Wu,^[a]^ Yadong Xu,^[a]^ Hongyue Wang,*^[a]^ Hongqiang Wang*^[a]^

^[a]^ State Key Laboratory of Solidification Processing, Center for Nano Energy Materials, School of Materials Science and Engineering, Northwestern Polytechnical University and Shaanxi Joint Laboratory of Graphene (NPU), Xi’an 710072, P. R. China.

*E-mails: hongyue.wang@nwpu.edu.cn, hongqiang.wang@nwpu.edu.cn

**Materials and Methods**

**Materials**

Lead bromide (PbBr_2_, 99.999%), cesium bromide (CsBr, 99.999%), Antimony bromide (SbBr_3_, 99.5%), Gallium bromide (GaBr_3_, 99.5%), lithium fluoride (LiF, 99.999%), anhydrous dimethyl sulfoxide (DMSO), diethyl ether (DE), and Polyvinylpyrrolidone (PVP) were purchased from Sigma-Aldrich. Phenylethylammonium bromide (PEABr) was purchased from Xi’an Polymer Technology Corp. β-Alanine (>98%) was obtained from Tokyo Chemical Industry (TCI). 2,2′,2″-(1,3,5-Benzinetriyl)-tris(1-phenyl-1-hbenzimidazole) (TBPi, 99.5%) was purchased from Luminescence Technology Corp. All the materials were directly used without further purification.

**Precursor solution preparation**

The quasi-2D perovskite PEA_2_(CsPbBr_3_)_2_PbBr_4_ precursor solutions were prepared by mixing solution A (PEA_2_PbBr_4_/DMSO) with solution B (CsPbBr_3_/DMSO) in a volume ratio of 2:1. Then, β-alanine was added to the PEA_2_(CsPbBr_3_)_2_PbBr_4_ precursor solution under continuous stirring for 2 h at 80 °C in ambient conditions. Solution A and B were prepared by dissolving appropriately stoichiometric CsBr, PEABr, XBr_3_ (X=Sb or Ga), and PbBr_2_ in DMSO under continuous stirring for 3 h in ambient conditions.

**Quasi-2D perovskite films deposition and characterization**

The quasi-2D perovskite films, with and without trivalent ions incorporation, were deposited by spin-coating at 3000 rpm and treated at 80 °C for 60 s under ambient conditions with a controlled relative humidity of 20-25% and a temperature of 20-30 °C. The PL and absorption spectra were collected using an Edinburgh FLS 980 spectrometer and a PerkinElmer Lambda 35 UV-vis-NIR spectrometer, respectively. Top-view scanning electron microscopy (SEM) images of perovskite films were taken with a field emission scanning electron microscope (FEI Nova) in secondary electron mode. A cross-sectional transmission electron microscopy (TEM) image of PeLEDs was obtained using an FEI Talos F200X equipped with a field emission gun (FEG) operated at 300 kV. Atomic force microscopy (AFM) measurements were recorded on a Bruker Dimension Icon atomic force microscope operated in air. X-ray photoelectron spectroscopy (XPS) and ultraviolet photoelectron spectrometer (UPS) were performed using a PHI Versa Probe II XPS system. XRD patterns were obtained using an X’Pert Pro MPD equipped with a diffraction beam monochromator and a Cu K X-ray source. The TRPL spectra of quasi-2D perovskite films were collected by the HORIDA DeltaFlex lifetime system. PLQY was measured using the C11347 quantum yield measurement system from Hamamatsu Photonics Trading (China) Co., Ltd. TA spectra were collected using a femtosecond (fs) pump-probe spectroscopy setup with a pump pulse of 410 nm, 1 kHz, and 356 uW. Temperature-dependent PL measurements were carried out by an RTS2 micro-fluorescence system equipped with a THMS600 temperature-control stage purchased from ZOLIX INSTRUMENTS CO., LTD.

**Device fabrication and characterization**

The patterned ITO glass was sequentially cleaned with detergent, deionized water, acetone, and IPA for 30 min each. Then, the precleaned ITO glass substrates were dried with a nitrogen gun and treated via UV-ozone for 15 min. 0.07 ml PVP solution (2 mg/mL in IPA) was dropped on the ITO glass slides at a rate of 9000 rpm for 60 s, followed by annealing at 100 °C for 10 minutes in air. After cooling to room temperature, the as-prepared perovskite precursor solution was spin-coated onto the PVP film at a rate of 3000 rpm for 70 s. After spin-coating for 30 s, 200 μL of DE was deposited onto the wet perovskite film. The wet film was then annealed at 80 °C for 1min to remove the residual solvent in ambient conditions. After cooling to room temperature, the perovskite films were transferred into the glove box for the following steps. Finally, TBPi (40 nm), LiF (1.5 nm), and Al (100 nm) bilayer cathodes were successively deposited using thermal evaporation equipment through a shadow mask under a high vacuum (<5×10^-4^ Pa). The active area of each tested devices was 4 mm^2^, as defined by the overlap between the patterned bottom ITO electrode and top metal electrode. All device characterizations were measured in a glovebox filled with a nitrogen atmosphere at room temperature (∼25 °C). The J-V-L curve, EQE-J curve, and EQE-luminance curve were obtained using the CDS 2600 EQE test system from Shanghai Labsphere Optical Equipment Co., Ltd. The test system is equipped with an integrating sphere, a Source-Meter (Keithley 2400), and a spectrometer (CDS2600). The device’s light emission is collected on top of the integration sphere, in line with the standard OLED characterization method.

**Computational method**

All the density functional theory (DFT) calculations were carried out using the Vienna Ab initio Simulation Package (VASP 5.4.4)(50,51). The exchange-correlation effects were handled using the generalized gradient approximation (GGA)(52) of the Perdew-Burke Ernzerhof (PBE)(53). The interactions between core and valence electrons were described by the projector augmented wave (PAW) method(54). The structure relaxations were performed with 450 eV plane-wave cutoff energy. The convergence criteria for supercell optimization were set to 10^-4^ eV for energy and 0.02 eV Å^−1^ for force, respectively.

The formation energy change ($\Delta E_{f}$) was determined as follows:

$$\Delta E_{f}=E\left( CBP-CNH \right)-E\left( CBP \right)-nE\left( CNH \right)+nE(Cs)$$

1. Kresse, G.; Furthmüller, J., Efficiency of ab-initio total energy calculations for metals and semiconductors using a plane-wave basis set. Computational Materials *Science* **1996,** *6* (1), 15-50.

2. Kresse, G.; Furthmüller, J., Efficient iterative schemes for ab initio total-energy calculations using a plane-wave basis set. *Physical Review B* **1996,** *54* (16), 11169-11186.

3. Perdew, J. P.; Burke, K.; Ernzerhof, M., Generalized Gradient Approximation Made Simple. *Physical Review Letters* **1996,** *77* (18), 3865-3868.

4. Perdew, J. P.; Ernzerhof, M.; Burke, K., Rationale for mixing exact exchange with density functional approximations. *The Journal of Chemical Physics* **1996,** *105* (22), 9982-9985.

5. Kresse, G.; Joubert, D., From ultrasoft pseudopotentials to the projector augmented-wave method. *Physical Review B* **1999,** *59* (3), 1758-1775.


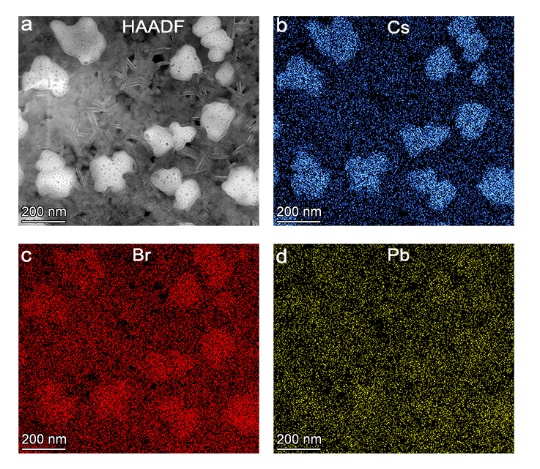


**Figure S1.** TEM-EDS analysis of the quasi-2D perovskite film.


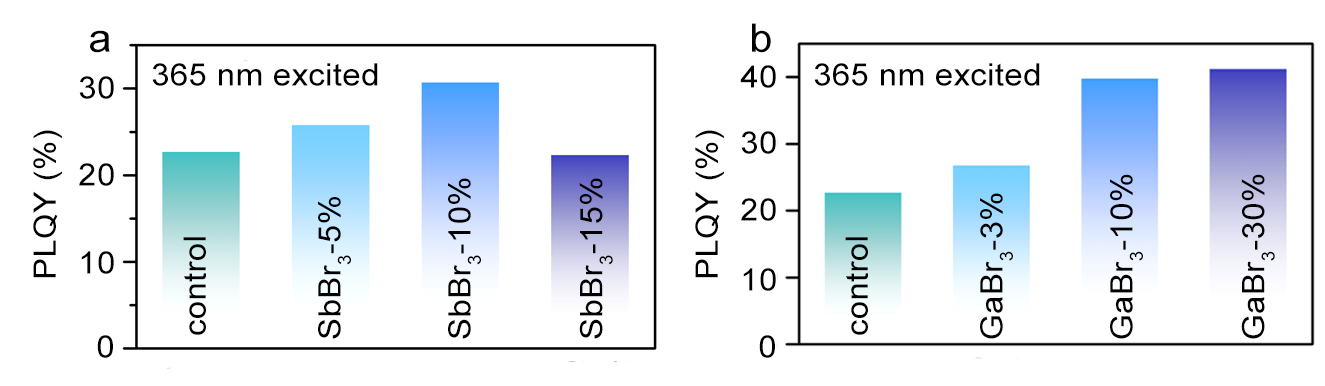


**Figure S2.** PLQY of perovskite films doped with (a) SbBr_3_ and (b) GaBr_3_.


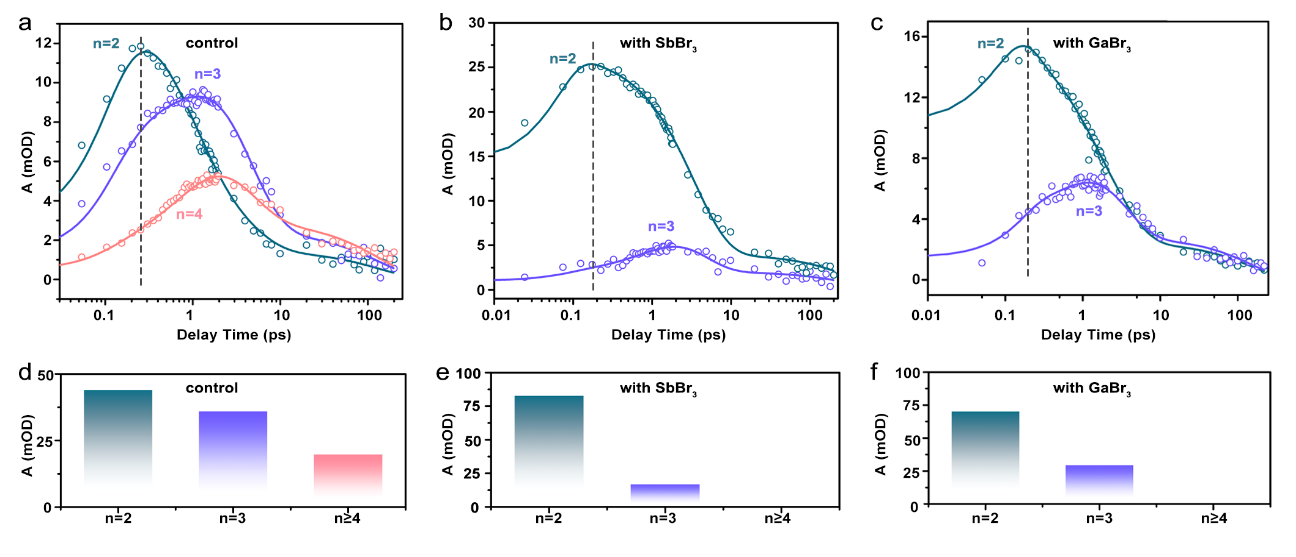


**Figure S3.** TA decay curves of (a) the control film, (b) SbBr_3_-treated perovskite film, and (c) GaBr_3_-treated perovskite film. Corresponding n phase distribution of (d) the control film, (e) SbBr_3_-treated perovskite film, and (f) GaBr_3_-treated perovskite film.





**Figure S4.** PL spectra of perovskite films formed with different PbBr_2_ contents.


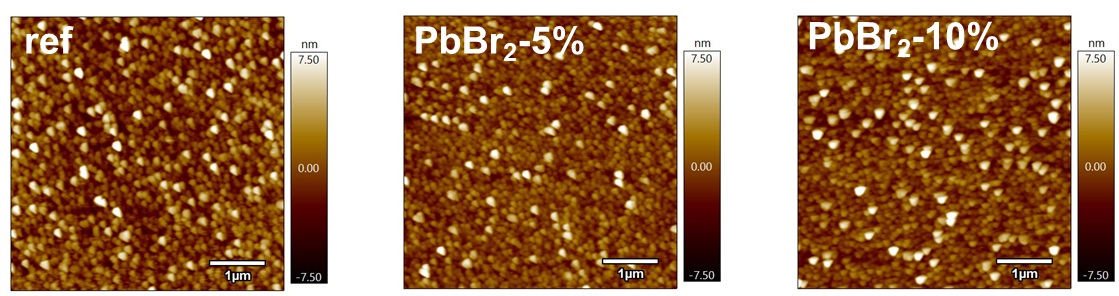


**Figure S5.** AFM images of perovskite films formed with different PbBr_2_ contents.


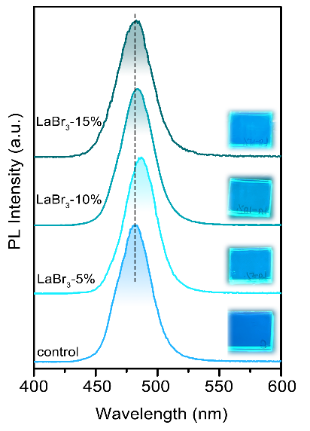


**Figure S6.** PL spectra of the control and LaBr₃-treated perovskite films.


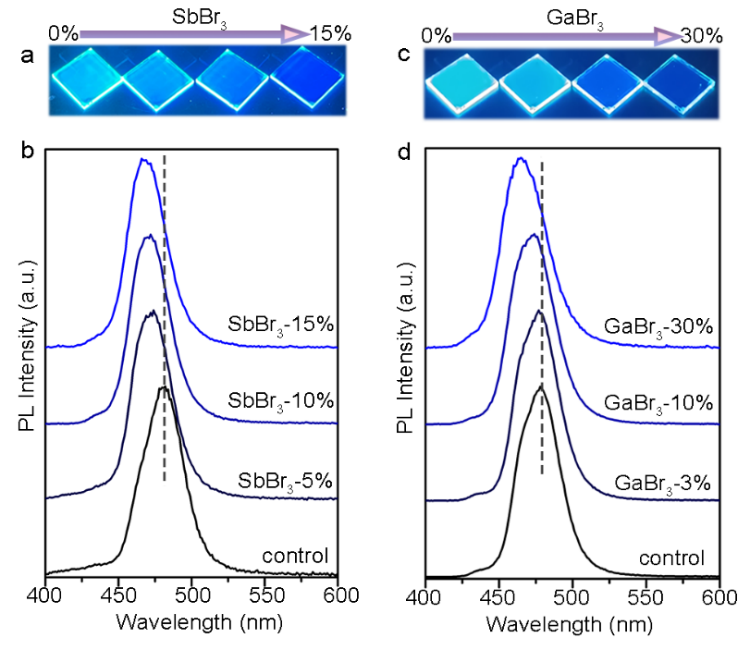


**Figure S7.** PL images under 365 nm UV light and the corresponding PL spectra excited with a 365 nm laser for perovskite films treated with different concentration of (a and b) SbBr_3_ and (c and d) GaBr_3_.


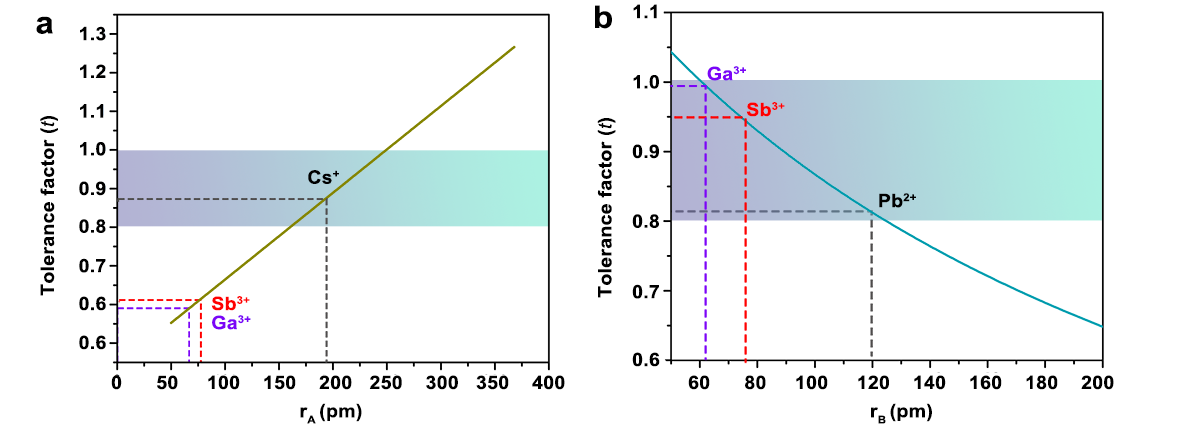


**Figure S8.** Tolerance factor (t) as a function of the (a) ‘A’ site cation radius (r_A_) and (b) ‘B’ site cation radius (r_B_) in the perovskite. The colored areas represent the suitable region for forming a perovskite structure at room temperature. The values for Cs^+^, Pb^2+^, Br^-^, Sb^3+^, and Ga^3+^ are marked in the plots. Previous studies suggest that metal cations with suitable ionic radii can substitute the “A” or “B” sites of the perovskite lattice. The effective ionic radii of Sb^3+^ and Ga^3+^ are 76 pm and 62 pm, respectively. Since their radii are much smaller than that of Cs^+^ (167 pm), incorporating these trivalent metal cations into “A” sites does not satisfy the tolerance factor criteria.


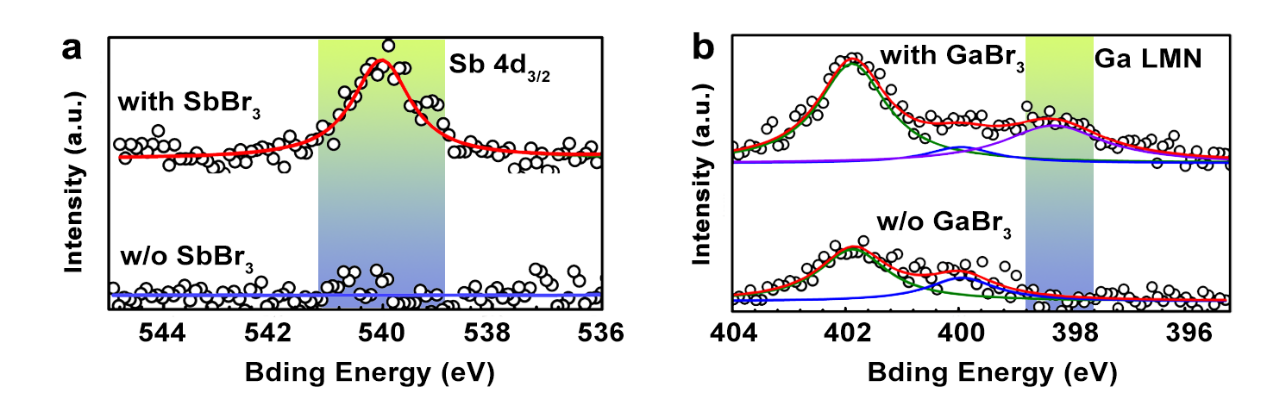


**Figure S9.** XPS spectra of perovskite films doped with (a) Sb^3+^ and (b) Ga^3+^.


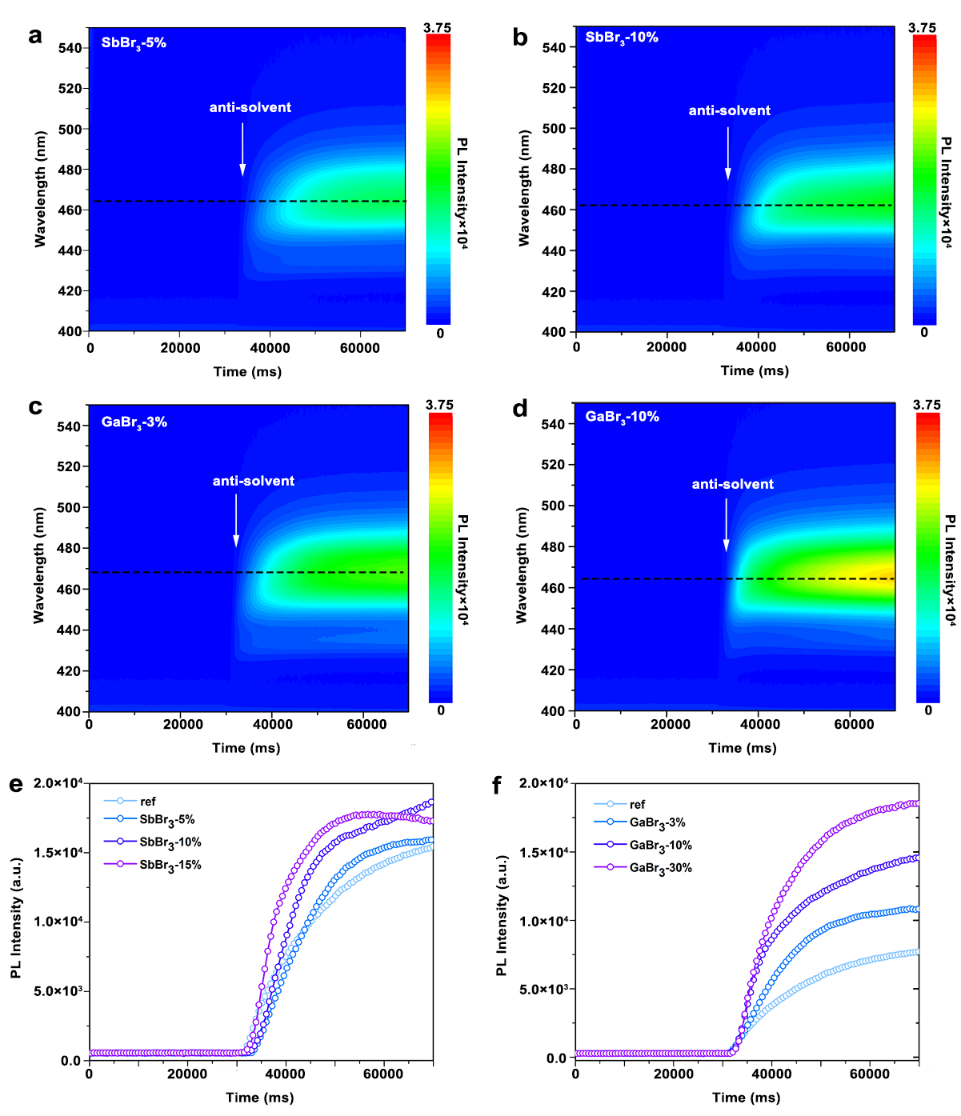


**Figure S10.** In-suit PL spectra of perovskite film doped with (a) 5% SbBr_3_, (b) 10% SbBr_3_, (c) 3% GaBr_3_, and (d)10% GaBr_3_. PL intensity as a function of spin-coating time for (e) SbBr_3_-treated and (f) GaBr_3_-treated perovskites films. The corresponding data were extracted from the in-suit PL spectra presented in Figure 3a-c and Figure S10a-d.


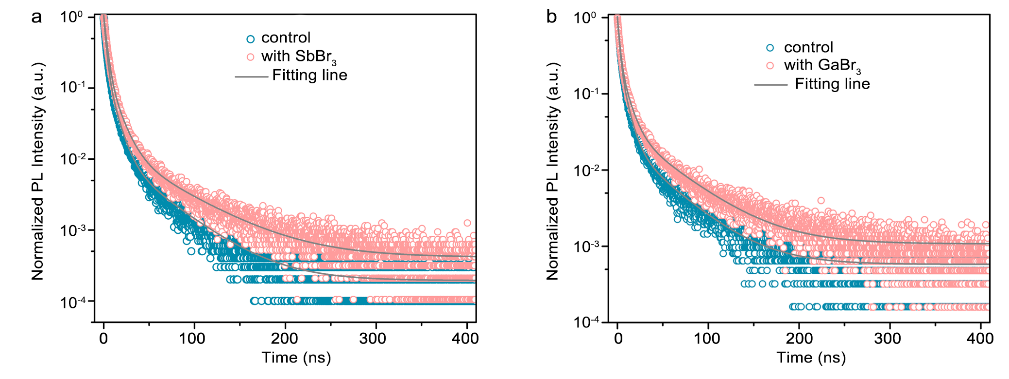


**Figure S11.** TRPL spectra of perovskite films doped with (a) SbBr_3_ and (b) GaBr_3_.


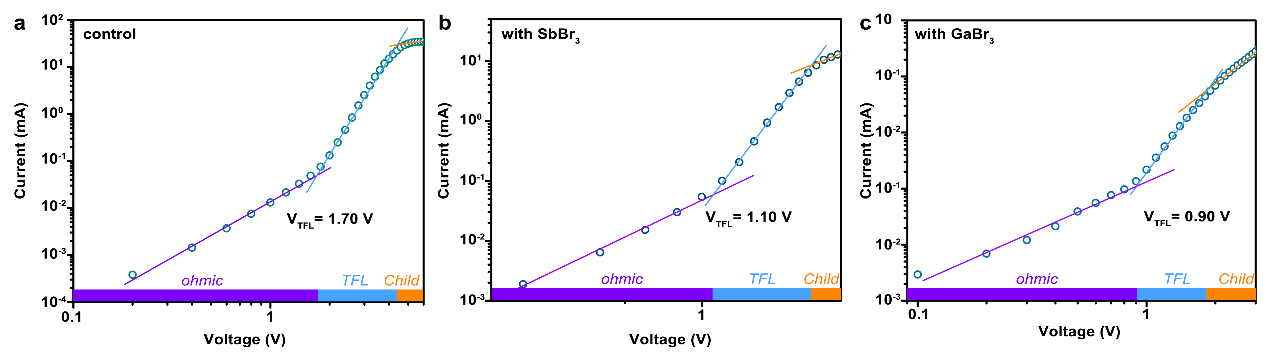


**Figure S12.** Space-charge-limited current measurements of (**a**) the control, (**b**) SbBr_3_-doped, and (**c**) GaBr_3_-doped perovskite films. The defect density ($N_{t}$) is determined by the formula: $N_{t}= \frac{{2V}_{TFL} \varepsilon\varepsilon_{0}}{eL^{2}}$, where $L$ is the thickness of the perovskite layer (~30 nm in this work), $e$is the elementary charge of the electron, $\varepsilon$ and $\varepsilon_{0}$ are the relative permittivity and the vacuum permittivity, respectively. The defect density of SbBr_3_- and GaBr_3_-doped perovskites film are estimated to be 1.34×10^17^ cm^-3^ and 1.10×10^17^ cm^-3^, respectively, which are lower than that of the control perovskite film (2.07×10^17^ cm^-3^).


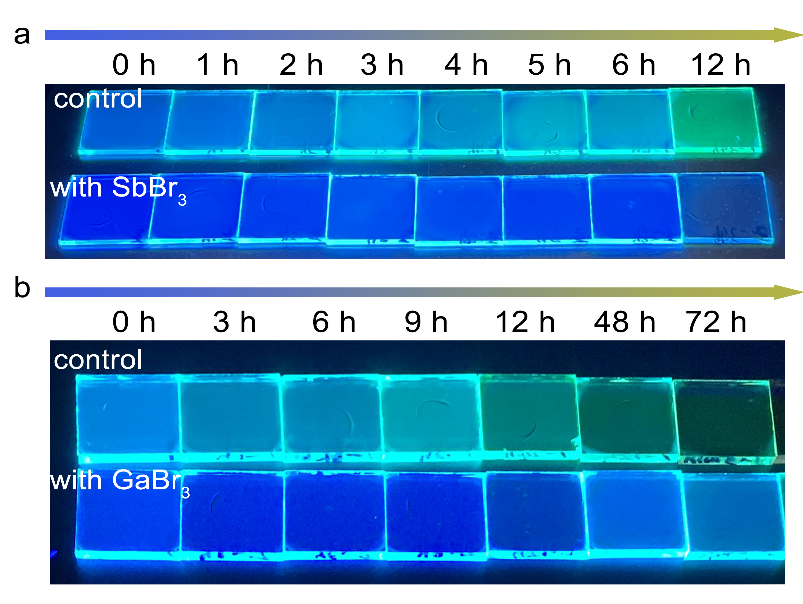


**Figure S13.** Photoluminescence image of (a) Sb^3+^-treated and (b) Ga^3+^-treated perovskite films under 70 ℃ within the timeframe of 72 h (under 365 nm UV light).


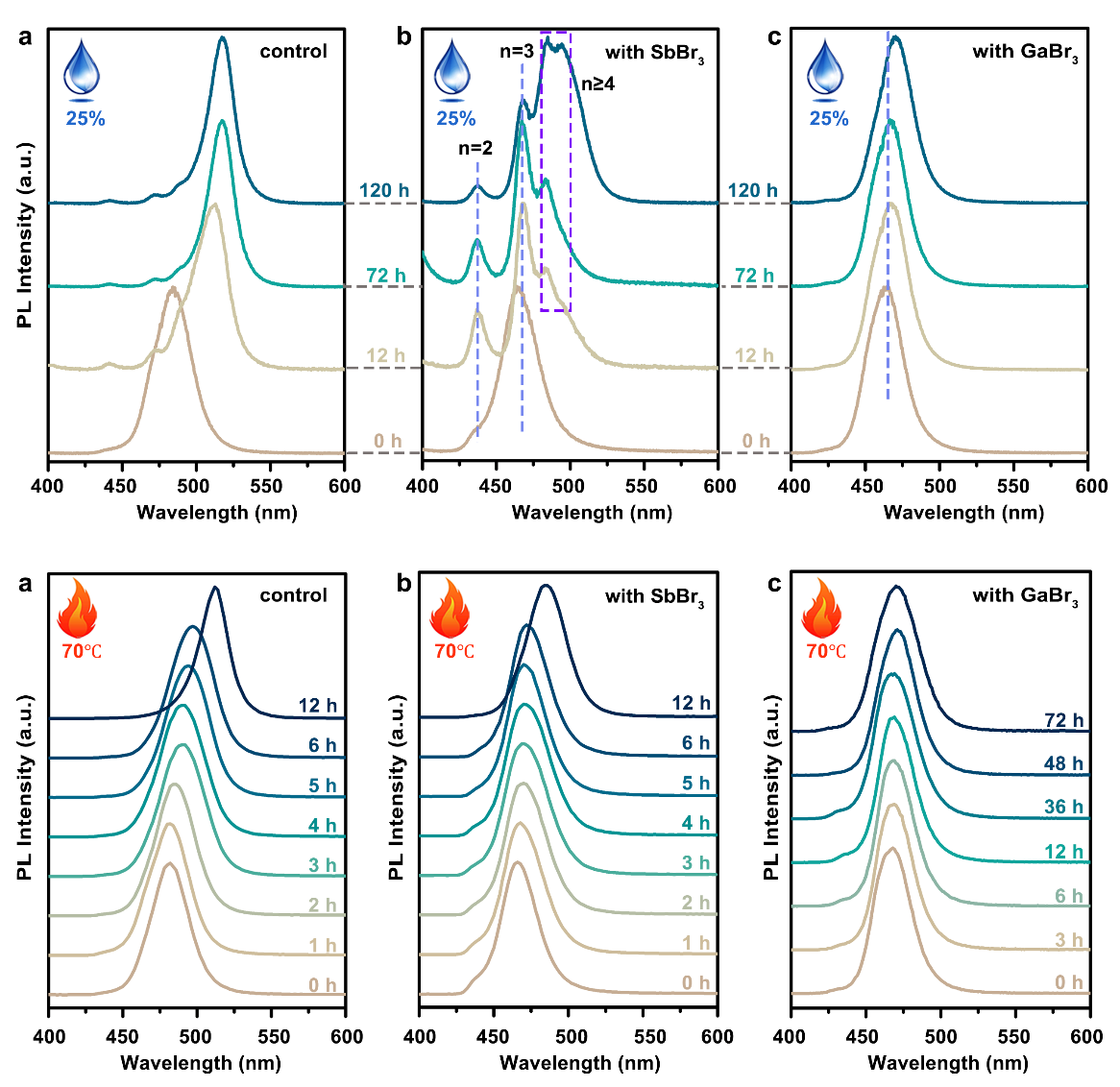


**Figure S14.** Thermal stability of (a) the control, (b) SbBr_3_-doped, and (c) GaBr_3_-doped films under 70 ℃ within the timeframe of 72 h.


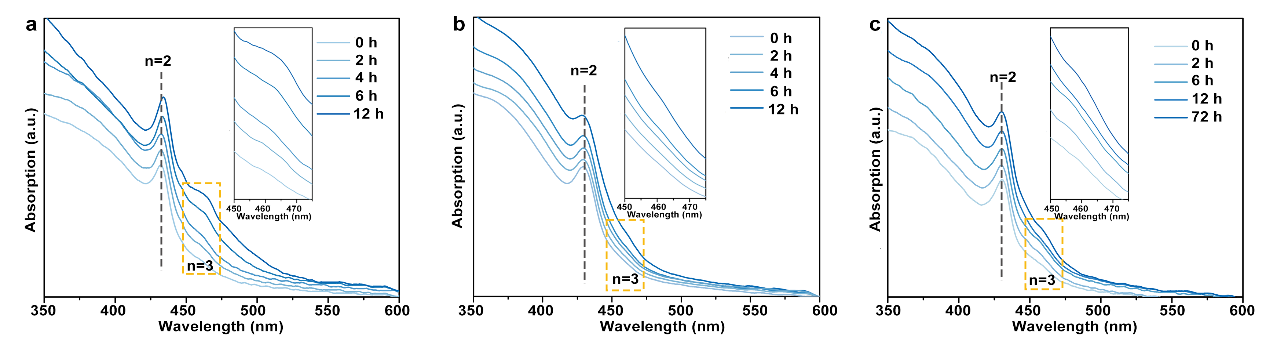


**Figure S15.** Absorption spectra of (a) the control, (b) SbBr_3_-doped, and (c) GaBr_3_-doped perovskite films at 70 ℃.


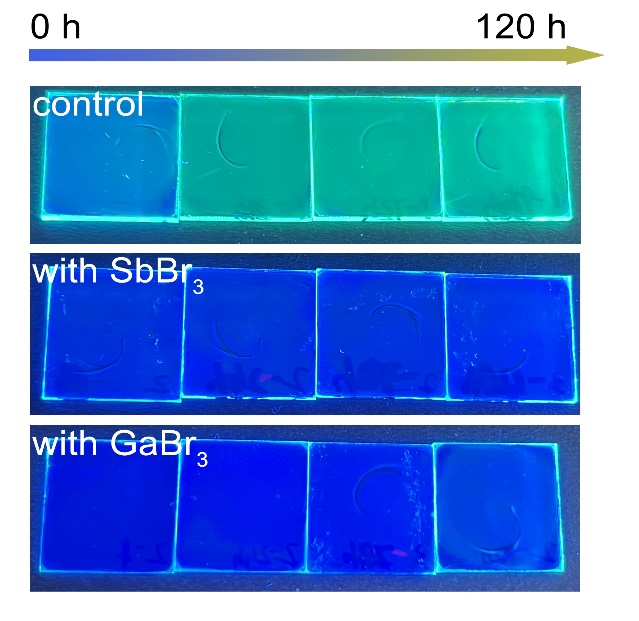


**Figure S16.** Image of control, Sb^3+^-treated, and Ga^3+^-treated perovskite films in an ambient environment with a relative humidity of 20%-25% within the timeframe of 120 h (under 365 nm UV light).


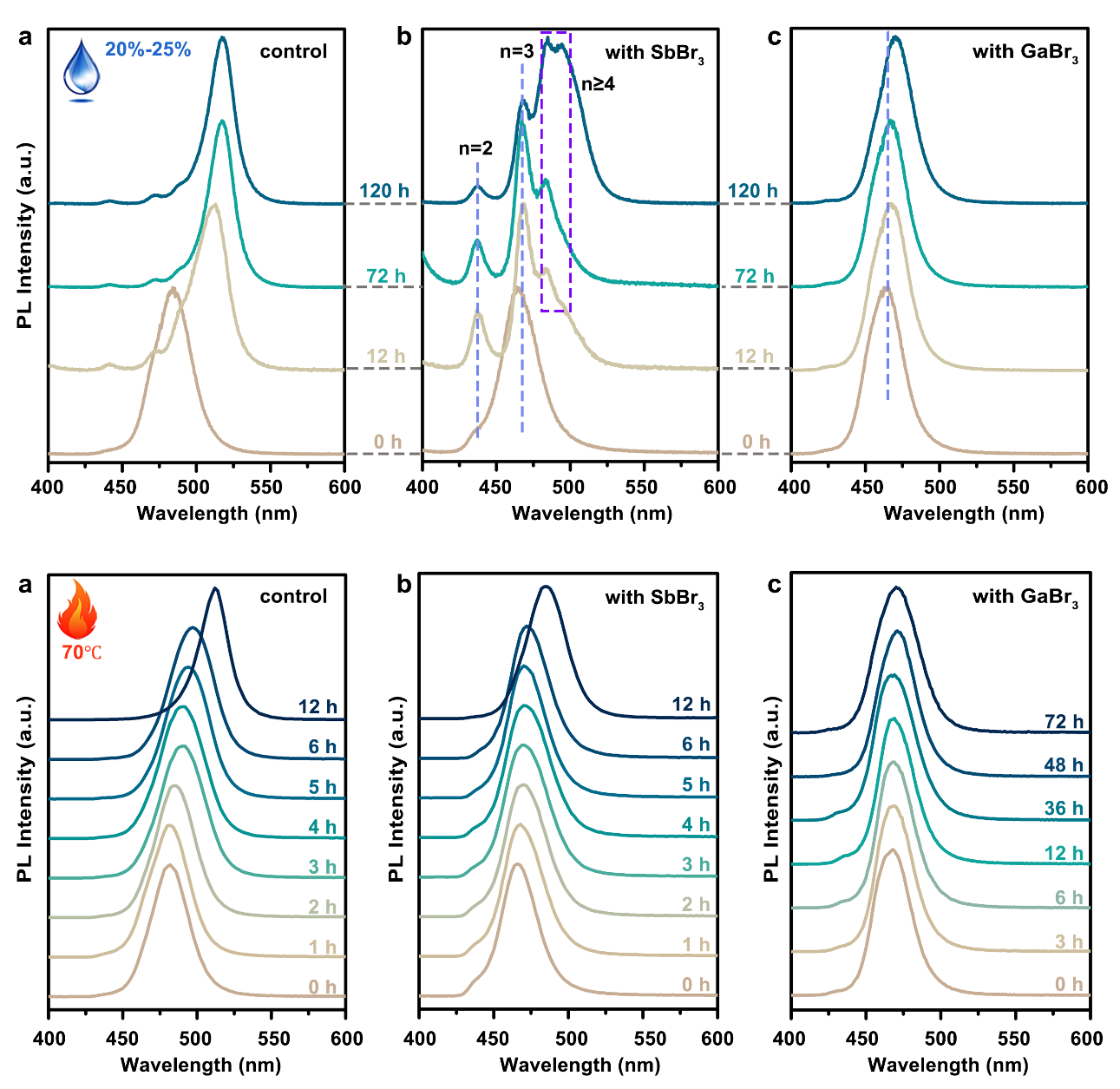


**Figure S17.** Evolution of PL spectra for (a) control, (b) Sb^3+^-doped, and (c) Ga^3+^-doped perovskite films in an ambient environment with a relative humidity of 20%-25% within the timeframe of 120 h.


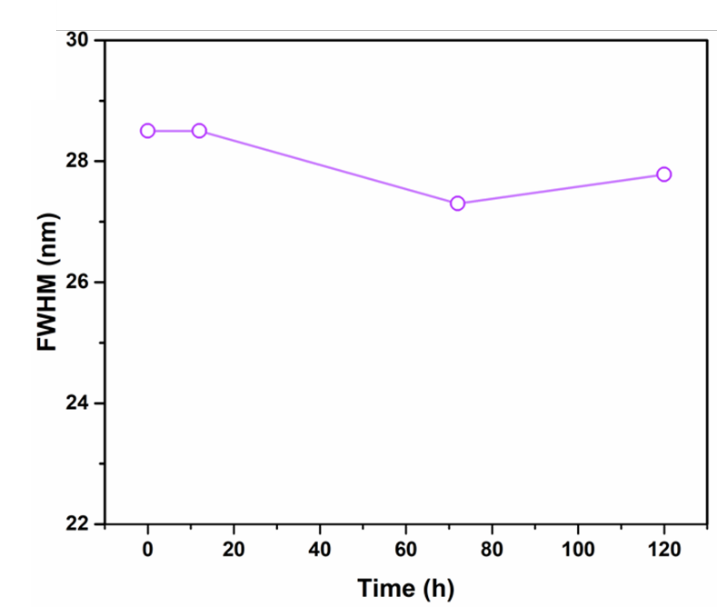


**Figure S18.** Evolution of the FWHM of the Ga^3+^-doped perovskite film in an ambient environment with a relative humidity of 20%-25% within the timeframe of 120 h.


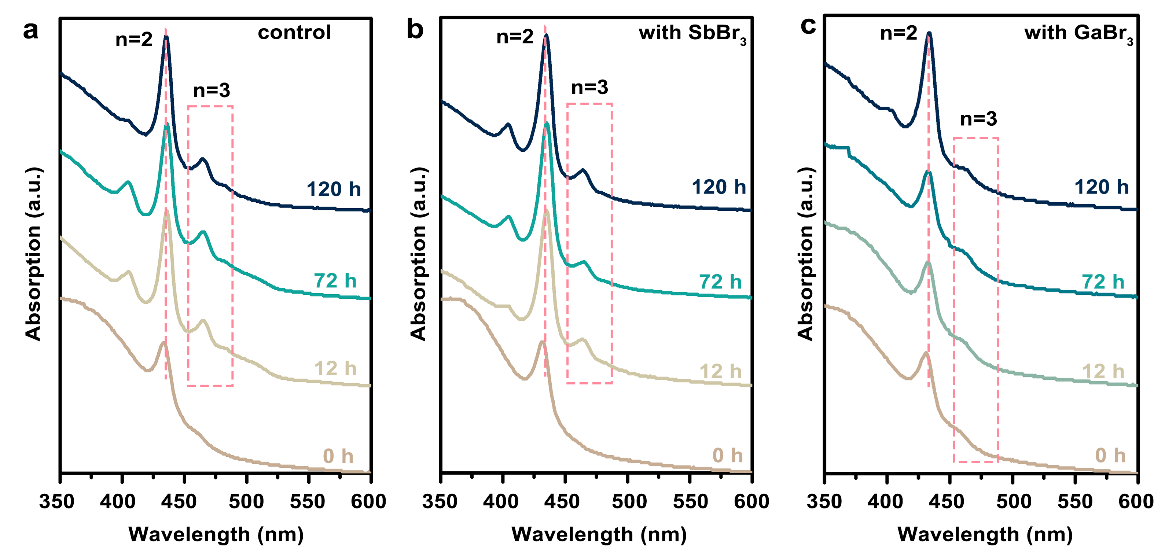


**Figure S19.** Absorption spectra of (a) control, (b) Sb^3+^-doped, and (c) Ga^3+^-doped perovskite films in an ambient environment with a relative humidity of 20%-25% within the timeframe of 120 h.


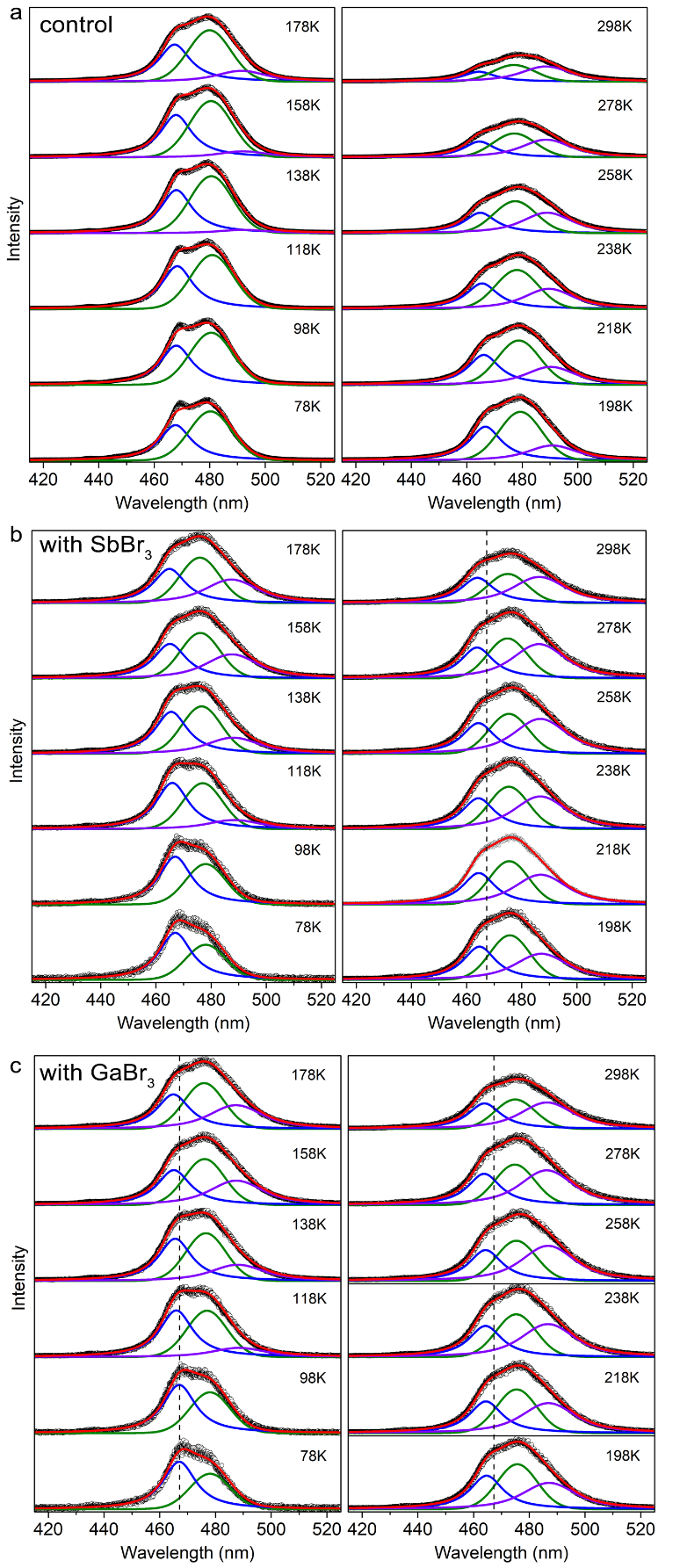


**Figure S20.** Fitting of temperature-dependent PL spectra for (a) the control film, (b) Sb^3+^-doped film, and (c) Ga^3+^-doped film.

**Supplementary note S1. energy barrier between two n phase in quasi-2D perovskite**

$n_{s}\underset{\to}{k} n_{l}$ (1)

where $n_{s}$ is the small n phase, $n_{l}$ is the large n phase, and $k$ is the rate constant of carrier transport from small n phase to large n phase. At a specific time, the relationship between the carrier concentration at the large n and small n phase can be quantified by the following equation:

$\frac{I_{n_{l}}}{I_{n_{s}}}=\frac{1-e^{-kt}}{e^{-kt}}=e^{kt}-1$ (2)

$\ln\frac{I_{n_{l}}}{I_{n_{s}}}=kt$ (3)

$k=\frac{1}{t}\left( \ln\frac{I_{n_{l}}}{I_{n_{s}}} \right)$ (4)

where $I_{n_{l}}$ and $I_{n_{s}}$are the PL integrated intensity of large n phase and small n phase, respectively. The equation of the Arrhenius equation is given by:

$k=A e^{\frac{-E_{a}}{RT}}$ (5)

where $k$ is rate constant, $A$ is Arrhenius constant (pre-exponential factor), $E_{a}$ is activation energy, $R$ is ideal gas constant and $T$ is reaction temperature. In consideration of the system under scrutiny operating at the microscopic level, it is appropriate to employ the $k_{b}$ as a substitute for the $R$, and the modified equation is determined by:

$k=A e^{\frac{-\acute{E_{a}}}{k_{b}T}}$ (6)

where $k_{b}$ is Boltzmann constant and $\acute{E_{a}}$represents the activation energy per individual molecule. Considering that the reaction rate constant for a given system remains constant under identical conditions, the following equations are consequently derived:

$A e^{\frac{-\acute{E_{a}}}{k_{b}T}}=\frac{1}{t}\left( \ln\frac{I_{n_{l}}}{I_{n_{s}}} \right)$ (7)

$\ln A-\frac{-\acute{E_{a}}}{k_{b}T}=\ln\left( \frac{1}{t} \right)+\ln\left( \ln\frac{I_{n_{l}}}{I_{n_{s}}} \right)$ (8)

Our analysis is based on the premise that the reaction achieves equilibrium at specified temperatures, thereby justifying the consideration of time as a constant parameter. The following equations are consequently derived:

$\ln\left( \ln\frac{I_{n_{l}}}{I_{n_{s}}} \right)=-\frac{\acute{E_{a}}}{k_{b}T}+C$ (9)

where $C$represents constant.


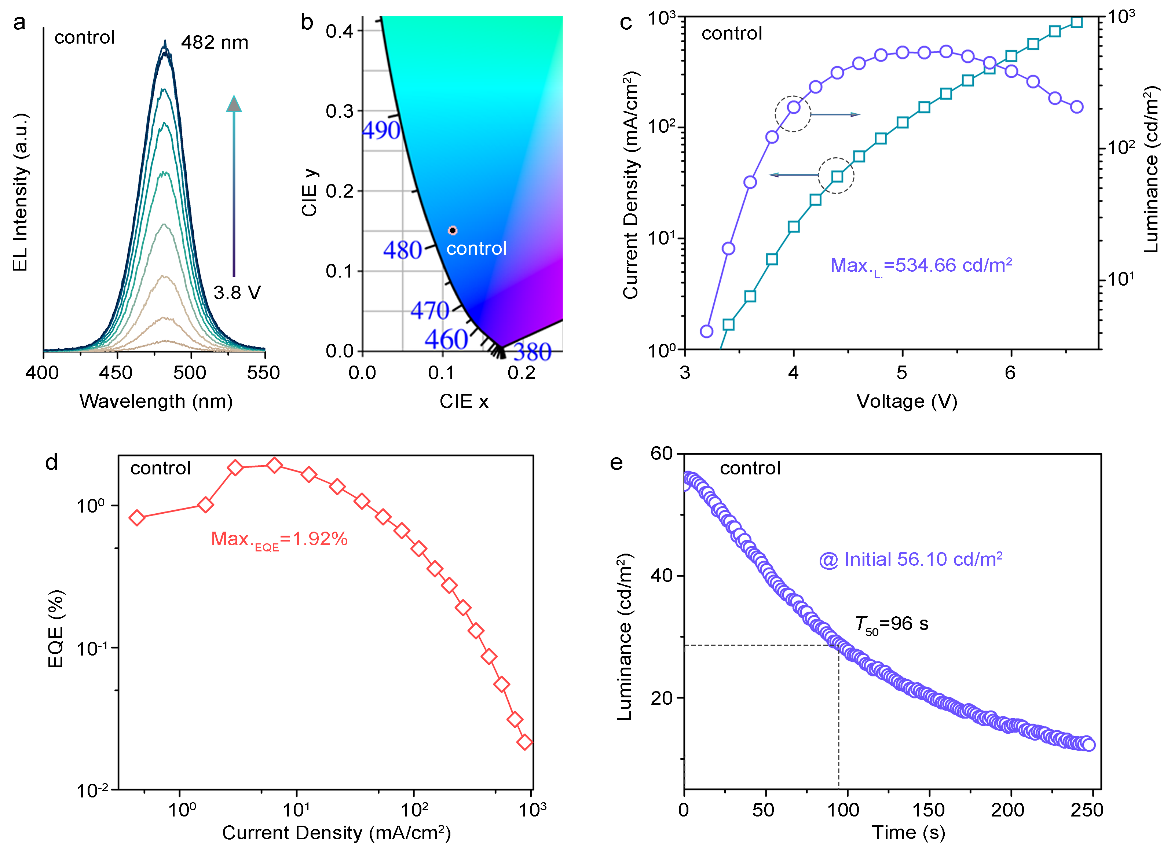


**Figure S21.** Performance of the control device. (a) EL spectra. (b) CIE coordinates. (c) Current density-voltage and luminance-voltage curves. (d) EQE-current density curve. (e) Operating lifetime measurement.


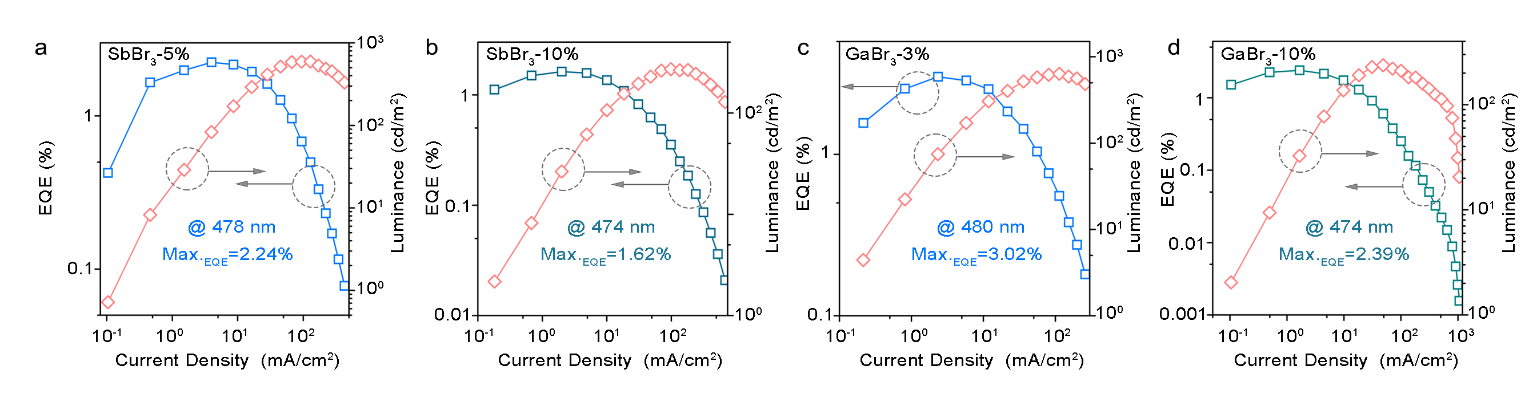


**Figure S22.** Current density-voltage and luminance-voltage curves of PeLEDs incorporated with (a) 5% SbBr_3_, (b) 10% SbBr_3_, (c) 3% GaBr_3_ and (d) 10% GaBr_3_.


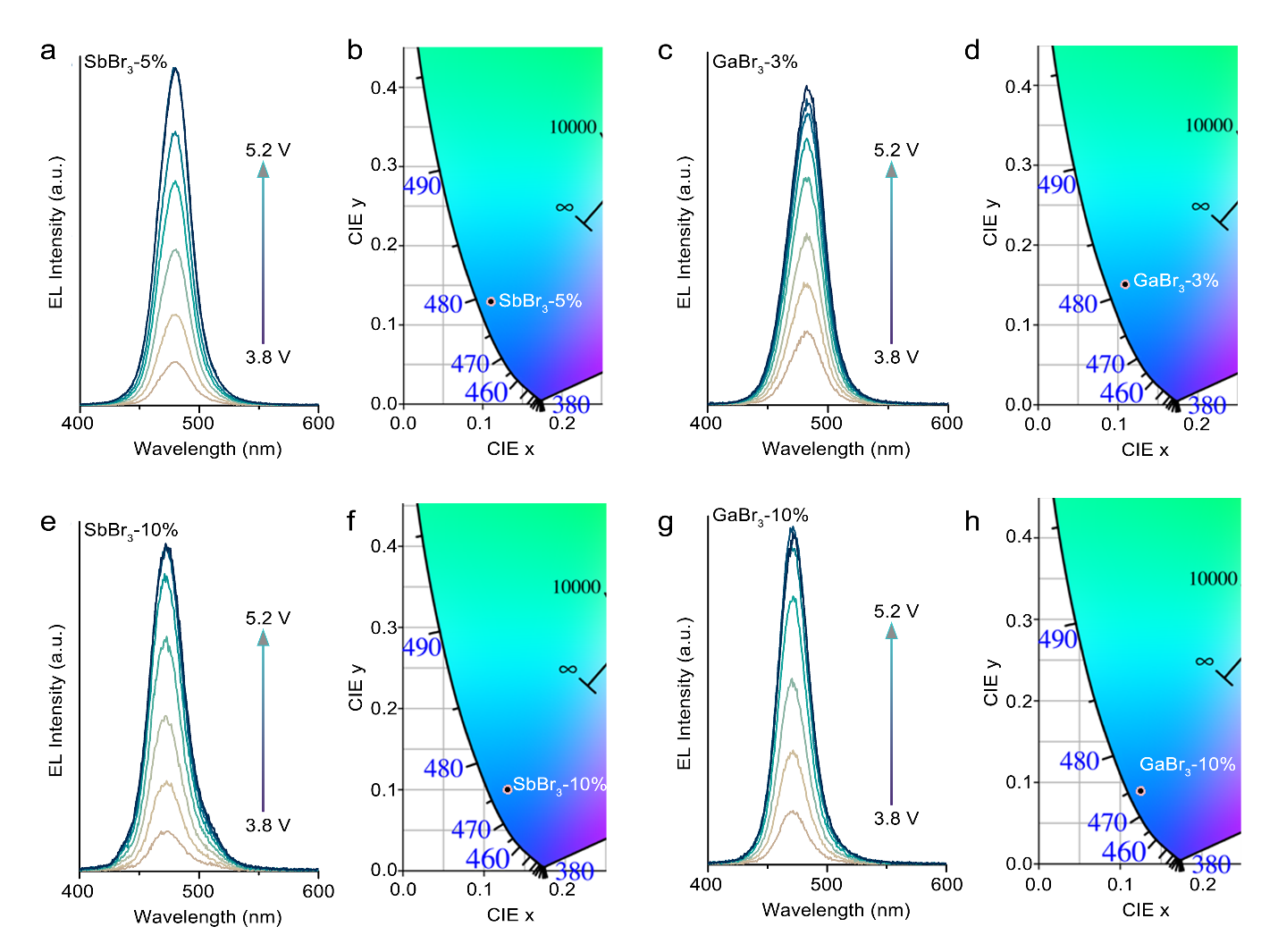


**Figure S23.** EL spectra at different bias voltages and the corresponding CIE coordinates of PeLEDs doped with (a and b) 5% SbBr_3_, (c and d) 3% GaBr_3_, (e and f) 10% SbBr_3_, and (g and h) 10% GaBr_3_.


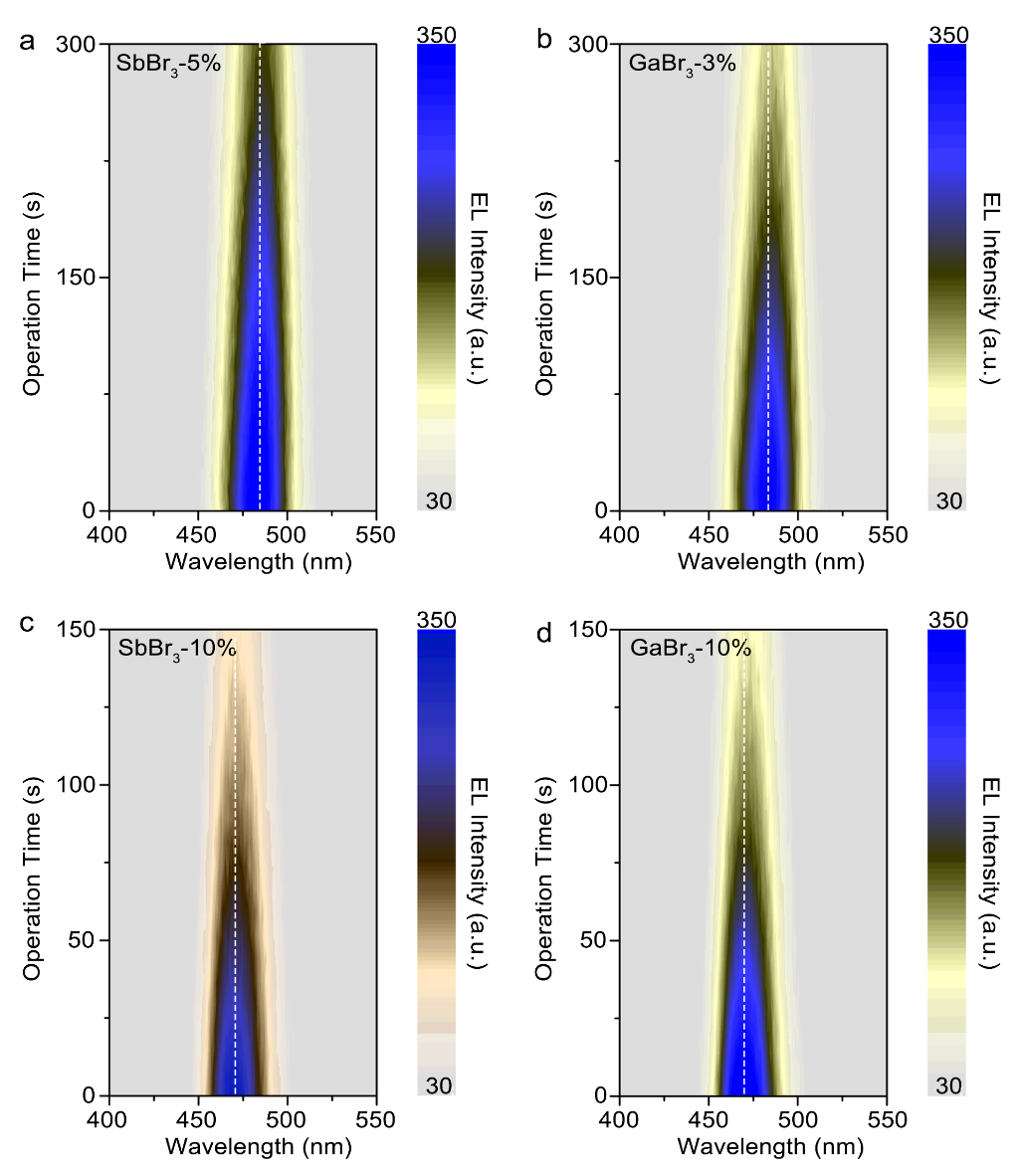


**Figure S24.** Time-dependent evolution of the EL spectrum for devices treated with (a) 5% SbBr_3_, (b) 3% GaBr_3_, (c) 10% SbBr_3_, and (d) 10% GaBr_3_.


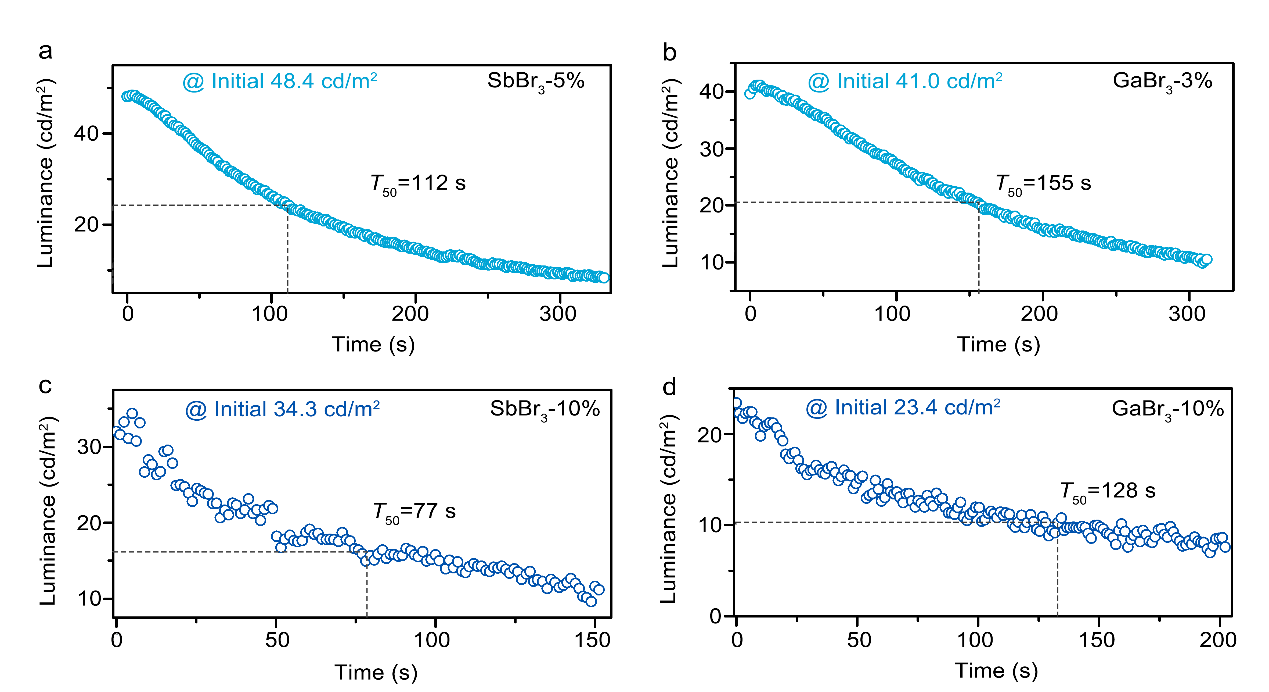


**Figure S25.** Operating lifetime of devices treated with (a) 5% SbBr_3_, (b) 3% GaBr_3_, (c) 10% SbBr_3_, and (d) 10% GaBr_3_ under nitrogen at room temperature.


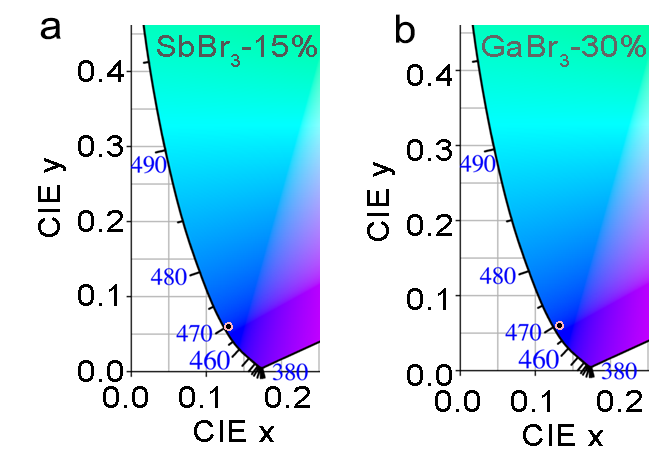


**Figure S26.** CIE coordinates of PeLEDs doped with (a) 15% SbBr_3_ and (b) 30% GaBr_3_.


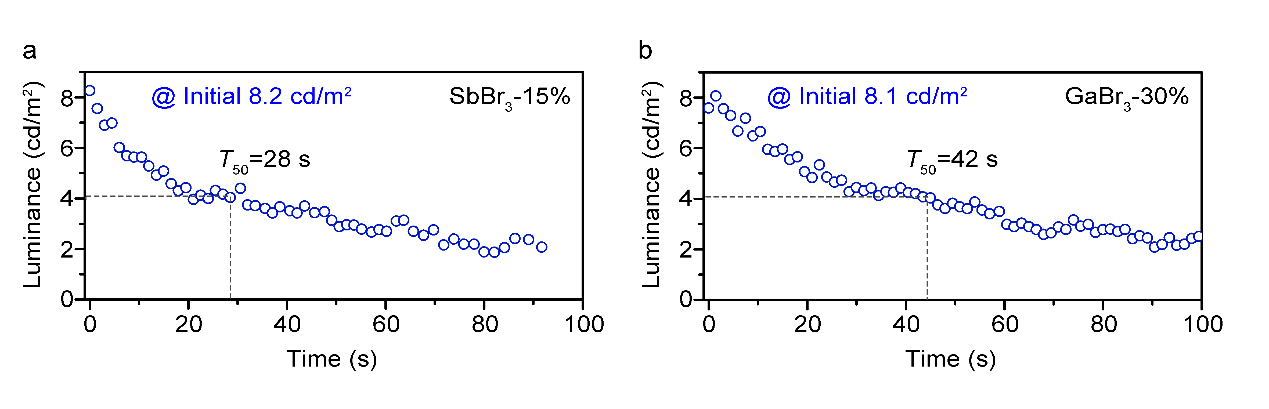


**Figure S27.** Operating lifetime of PeLEDs incorporated with (a) 15% SbBr_3_ and (b) 30% GaBr_3_ under nitrogen at room temperature.

**Table S1** The parameters of carrier lifetime by fitting the TRPL spectra based on control and SbBr_3_-treated perovskite films.

| Sample | A_1_ (%) | τ_1_ (ns) | A_2_ (%) | τ_2_ (ns) | A_3_ (%) | τ_3_ (ns) | τ_ave_ (ns) |
| --- | --- | --- | --- | --- | --- | --- | --- |
| control | 84.06 | 2.41 | 26.16 | 7.21 | 2.46 | 40.16 | 11.78 |
| with SbBr_3_ | 85.94 | 3.18 | 18.5 | 10.83 | 1.53 | 55.69 | 13.93 |

**Table S2** The parameters of carrier lifetime by fitting the TRPL spectra based on control and GaBr_3_-treated perovskite films.

| Sample | A_1_ (%) | τ_1_ (ns) | A_2_ (%) | τ_2_ (ns) | A_3_ (%) | τ_3_ (ns) | τ_ave_ (ns) |
| --- | --- | --- | --- | --- | --- | --- | --- |
| control | 73.83 | 2.16 | 26.16 | 7.21 | 2.46 | 40.16 | 12.69 |
| with GaBr_3_ | 70.78 | 2.22 | 27.13 | 8.35 | 3.75 | 45.78 | 18.19 |

**Table S3** Parameters derived via fitting the temperature dependence of the PL FWHM with Boson model.

| Perovskite films | Γ_inh_ (meV) | Γ_LO_ (meV) | Γ_EO_ (meV) |
| --- | --- | --- | --- |
| control | 139.45 | 160.23 | 19.68 |
| SbBr_3_-5% | 106.15 | 115.31 | 13.98 |
| GaBr_3_-10% | 125.97 | 96.30 | 12.37 |

The variation of FWHM was fitted by Boson model:

$$\Gamma\left( T \right)= \Gamma_{inh} +\sigma T+\frac{\Gamma_{LO}}{e^{{E_{LO}}/{{(k}_{B}T)}}-1}$$

where $\Gamma_{inh}$ is linewidth of zero-phonon emission at zero-temperature, which is associated with the inhomogeneous broadening, $\sigma$ is an exciton-acoustic phonon coupling coefficient, $\Gamma_{LO}$ represents the exciton longitudinal optical (LO) phonon coupling coefficient, $E_{LO}$ is the LO phonon energy, $k_{B}$ denotes Boltzmann constant and T is temperature. From 80 K to room temperature, the electron-phonon interaction is dominated by LO phonon, and the influence of acoustic phonon is negligible. We thus defined $\sigma$ = 0 for the fitting in this work, and the fitting parameters are shown in this Table S1.
